# The Mating Type Locus (*MAT*) and Sexual Reproduction of *Cryptococcus heveanensis*: Insights into the Evolution of Sex and Sex-Determining Chromosomal Regions in Fungi

**DOI:** 10.1371/journal.pgen.1000961

**Published:** 2010-05-20

**Authors:** Banu Metin, Keisha Findley, Joseph Heitman

**Affiliations:** Department of Molecular Genetics and Microbiology, Duke University Medical Center, Durham, North Carolina, United States of America; University of California San Francisco, United States of America

## Abstract

Mating in basidiomycetous fungi is often controlled by two unlinked, multiallelic loci encoding homeodomain transcription factors or pheromones/pheromone receptors. In contrast to this tetrapolar organization, *Cryptococcus neoformans/Cryptococcus gattii* have a bipolar mating system, and a single biallelic locus governs sexual reproduction. The *C. neoformans MAT* locus is unusually large (>100 kb), contains >20 genes, and enhances virulence. Previous comparative genomic studies provided insights into how this unusual *MAT* locus might have evolved involving gene acquisitions into two unlinked loci and fusion into one contiguous locus, converting an ancestral tetrapolar system to a bipolar one. Here we tested this model by studying *Cryptococcus heveanensis*, a sister species to the pathogenic *Cryptococcus* species complex. An extant sexual cycle was discovered; co-incubating fertile isolates results in the teleomorph (*Kwoniella heveanensis*) with dikaryotic hyphae, clamp connections, septate basidia, and basidiospores. To characterize the *C. heveanensis MAT* locus, a fosmid library was screened with *C. neoformans/C. gattii MAT* genes. Positive fosmids were sequenced and assembled to generate two large probably unlinked *MAT* gene clusters: one corresponding to the homeodomain locus and the other to the pheromone/receptor locus. Strikingly, two divergent homeodomain genes (*SXI1, SXI2*) are present, similar to the bE/bW *Ustilago maydis* paradigm, suggesting one or the other homeodomain gene was recently lost in *C. neoformans/C. gattii*. Sequencing *MAT* genes from other *C. heveanensis* isolates revealed a multiallelic homeodomain locus and at least a biallelic pheromone/receptor locus, similar to known tetrapolar species. Taken together, these studies reveal an extant *C. heveanensis* sexual cycle, define the structure of its *MAT* locus consistent with tetrapolar mating, and support the proposed evolutionary model for the bipolar *Cryptococcus MAT* locus revealing transitions in sexuality concomitant with emergence of a pathogenic clade. These studies provide insight into convergent processes that independently punctuated evolution of sex-determining loci and sex chromosomes in fungi, plants, and animals.

## Author Summary

Comparative genomics provides an approach to understand evolution of sex-determining regions in animals and fungi. One example is the unusual mating-type (*MAT*) locus of pathogenic *Cryptococcus* species (>100 kb, >20 genes). The fungal *MAT* locus is a model for gene cluster evolution and shares features with sex chromosomes of plants/animals. In previous studies with *C. neoformans/C. gattii*, a model for this unusual locus was proposed in which two ancestral unlinked loci were fused into one contiguous locus. *C. heveanensis* represents an evolutionary window to test this model, since it is related to but distinct from pathogenic *Cryptococcus* species. The organization of *MAT* in two probably unlinked loci in *C. heveanensis* provides evidence supporting the model and additional novel insights into how serial gene acquisitions created evolutionary strata. We also discovered an extant sexual cycle for *C. heveanensis* and correlated fertility with *MAT* alleles. These findings provide direct experimental validation of the central tenants of sex-chromosome evolution originally proposed by Ohno involving sex determinants arising on autosomes, formation of gene clusters with coherent functions in sex, and mechanisms by which specialized recombinationally suppressed genomic regions expand to capture an entire chromosome as a sex chromosome.

## Introduction

Although many organisms, in particular microorganisms, can reproduce both asexually and sexually, the vast majority appears to undergo sexual reproduction during their life cycles. The hypotheses advanced as to why sex is so ubiquitous are myriad, but center on several recurrent themes [1], [2]. First, the admixture of genetic material from two genetically distinct individuals that occurs during sexual reproduction may give rise to novel gene combinations that result in offspring better suited to novel or changing environments. Second, sexual reproduction may serve to remove deleterious mutations, such as transposons, that have arisen within the genome. Finally, sex might serve both roles, facilitating combinations of successful alleles and simultaneously purging the genome of deleterious ones.

Until recently it has been difficult to test these models for the possible benefits of sex in experimentally tractable model organisms. However, recent studies with the model yeast *S. cerevisiae* have provided direct experimental evidence for a benefit of sexual reproduction. Isogenic diploid strains were engineered that are either capable of sporulation with meiotic recombination (sexual) or were able to sporulate but not undergo meiotic recombination (asexual, *spo11*,*13* mutant) [3]. Under a variety of different stressful environments, the sexual strain had a competitive advantage compared to the asexual strain. A series of related studies in both *S. cerevisiae* and the model alga *Chlamydomonas* provide additional support for a benefit conferred by sex in response to novel or challenging environments [4]–[6]. In response to constant environments, asexual reproduction is also likely to be of relative benefit by relieving strains from the metabolic and genetic costs associated with sex [7]. Thus, a balance between sexual and asexual reproduction may be struck in response to different environmental conditions.

While the vast majority of sexually reproducing organisms occur as just two sexes or mating types, transitions in sexuality from two to multiple mating types, and vice versa, have occurred in the fungal kingdom. Sexual reproduction is common in fungi, and mating type occurs in two general patterns: bipolar and tetrapolar mating type (reviewed in [8]–[10]. In the bipolar systems, a single genetic locus known as the mating type locus (*MAT*) occurs in two alternate forms, known as idiomorphs (**a** or α, **a** or A, + or −, P or M), and these govern the identity of the cell [11]–[13]. Co-incubation of isolates of opposite mating type under suitable conditions leads to sexual reproduction. Species with bipolar mating systems are found in the ascomycete, basidiomycete, and zygomycete phyla, providing evidence that this is an ancestral organization. In the basidiomycete phylum, many species instead have a more complex sex determining system, known as tetrapolar, in which two unlinked sex determining loci are present [8], [10], [14], [15]. One locus encodes homeodomain transcription factors and the other locus encodes pheromones and pheromone receptors, and both loci must differ for sexual reproduction to occur. In many species, these loci are multiallelic, resulting in literally thousands of different mating types, which promotes outcrossing [16]. Transitions from tetrapolar to bipolar mating type determination have occurred multiple independent times, and in several examples result from fusions of the two unlinked loci to form one contiguous region (reviewed in [17], [18]), potentially illustrating common evolutionary pressures limiting mating-type/sexes to just two.

Although many fungi are currently classified as asexual, genomics has revealed that many such fungi retain the mating type locus and machinery necessary for both mating and meiosis [19]–[24]. In some cases, population genetics studies also reveal an equal distribution of opposite mating types in nature, and evidence for recombination [25]–[28]. In some notable recent examples, such as *Aspergillus parasiticus* and *Aspergillus fumigatus*, an extant sexual cycle has been discovered [29], [30]. Thus, there may be few truly asexual fungi, and many fungi, and perhaps most, for which cryptic sexual cycles remain to be discovered [31], [32].

Sexual reproduction in the basidiomycetous *Cryptococcus* pathogenic species complex involves a well-defined bipolar mating type system with two opposite mating types, **a** and α [33]–[38]. Cell-cell fusion leads to the production of dikaryotic hyphae with fused clamp connections, and ultimately the hyphal tips differentiate to form basidia in which nuclear fusion and meiosis occur. Budding from the basidial surface then produces long chains of basidiospores, the suspected infectious propagules [39], [40] for reviews see [41]–[43].

Molecular analysis of the *Cryptococcus* mating type locus reveals it to be unusual, spanning >120 kb and encompassing >20 genes [44]–[49]. With the exception of the homeodomain genes, *SXI1*α and *SXI2* **a**, which govern cell type identity, the **a** and α mating type alleles are otherwise composed of divergent alleles of a common gene set [50], [51]. A genomic approach comparing the extant, diverged mating type alleles in *C. neoformans* var. *grubii* (serotype A), *C. neoformans* var. *neoformans* (serotype D), and *C. gattii* revealed that the *MAT* locus is composed of four gene strata of distinct evolutionary ages, including an ancient set of ancestral genes, two gene strata of more intermediate evolutionary origin, and a set of apparently recently acquired genes that are species- but not mating type-specific [47]. The finding that the *Cryptococcus* bipolar *MAT* locus contains all of the genes normally found in the two unlinked tetrapolar loci suggests that a fusion of the two loci might have been involved in the origins of this unusual gene cluster linked to virulence and differentiation. The hypothesized evolutionary model posits that an ancestral tetrapolar mating system expanded by a series of acquisitions of genes of related function to form two large gene clusters that subsequently underwent fusion via a chromosomal translocation to form an unstable tripolar intermediate stage that subsequently underwent recombination or gene conversion to form the bipolar state. A series of more recent gene conversions and inversions then occurred, giving rise to the extant bipolar mating type alleles and resetting the molecular clock for some genes within distinct strata and even evicting genes from *MAT* in other examples [47]. The recent discovery that the *MAT* locus is flanked by and contains recombination hotspots provides evidence that the expansion and fusion of these loci may have been driven by activation of recombination [52]. In a recent study, the homeodomain genes were relocated to an unlinked genomic location, providing direct experimental support that *Cryptococcus* can be engineered to complete a tetrapolar sexual cycle and that the tripolar intermediate may have conferred an evolutionary disadvantage and therefore have been transitory [53]. A central goal of the studies presented here was to further rigorously examine the proposed *MAT* evolutionary model by comparative studies with related but divergent fungal species.

In a recent study, a multi-locus gene phylogeny was developed with species closely related to the pathogenic *Cryptococcus* species complex, which includes *C. neoformans* var. *neoformans*, *C. neoformans* var. *grubii*, and *C. gattii* [54]. In this study, the yeasts analyzed defined two clades. One contains the pathogenic species complex as well as *Cryptococcus amylolentus*, *Tsuchiyaea wingfieldii*, and *Filobasidiella depauperata*, and was termed the *sensu stricto* clade. The other clade encompasses yeasts more distantly related to the pathogenic species complex, and was termed the *sensu lato* group. *C. heveanensis* lies in the *sensu lato* clade, together with *Bullera dendrophila*, *Cryptococcus bestiolae*, *Cryptococcus dejecticola*, and a recently described sexual species, *Kwoniella mangroviensis* [55]. The fact that *C. heveanensis* is located in a sister clade to the pathogenic *Cryptococcus* species complex makes it an excellent candidate to provide insights into the evolution of the *MAT* locus and sexual reproduction in pathogenic *Cryptoccocus* species.

The closest yeast with a defined sexual cycle to *C. heveanensis* is *K. mangroviensis*, which is also located in the *sensu lato* clade containing yeasts more distantly related to the pathogenic *Cryptococcus* species [54]. The basidial structure of *K. mangroviensis* differs from that in *C. neoformans* and *C. gattii* and contains septa (phragmobasidia) whereas the pathogenic *Cryptococcus* species have nonseptate basidia (holobasidia) [33], [55]. When pairs of compatible *K. mangroviensis* strains are co-cultured on CMA medium, dikaryotic hyphae with clamp connections and basidia are produced [55]. Of 19 *K. mangroviensis* strains isolated from the Bahamas, 7 were fertile (37%) and 12 were sterile (63%). Among the seven fertile isolates, six were assigned to the α mating type and one to the A mating type. From these results, the authors proposed that the mating system appeared to be bipolar and biallelic. The nature of the *K. mangroviensis* mating system will be further clarified by analysis of additional fertile strains, progeny from genetic crosses, and the *MAT* locus.

In this study, we analyzed the mating behavior and *MAT* locus structure of *C. heveanensis*. First, we discovered an extant sexual cycle for *C. heveanensis* involving dikaryotic hyphae with clamp connections and basidia with cruciate septa and termed this teleomorph *Kwoniella heveanensis*. Second, we determined the structure of the *MAT* locus involving two probably unlinked gene clusters corresponding to the homeodomain locus and the pheromone/receptor locus. The presence of two divergently transcribed homeodomain genes in the homeodomain locus suggests this *MAT* locus arrangement is ancestral to the pathogenic *Cryptococcus* species, and one or the other homeodomain gene has been lost recently from *MAT* in *C. neoformans* and *C. gattii*. In addition, the presence of numerous other genes around the C. *heveanensis MAT* locus that correspond to genes located on the same chromosome but far away from *MAT* in *C. neoformans* suggests intrachromosomal rearrangements occurred during the evolution of the *C. neoformans MAT* locus and flanking regions. Characterizing *MAT* alleles from other *C. heveanensis* isolates suggests that the homeodomain locus is multiallelic and the pheromone/receptor locus is at least biallelic. Additionally, the *MAT*-specific region is likely to be restricted to the *SXI1* and *SXI2* genes in the homeodomain locus and spans at least the *STE3*, *STE12*, *MFA1/2*, *CNB00600*, and *CNG04540* genes in the pheromone/receptor locus, consistent with a tetrapolar mating system. This analysis reveals an evolutionary intermediate with two apparently unlinked *MAT* loci into which genes have been acquired, lost, and translocated, providing insights into the events that punctuated the evolution of a contiguous sex determining gene cluster in *C. neoformans* and *C. gattii*. These studies serve as an evolutionary window both for the evolution of mating type loci in fungi, and also into similar events that have driven the evolution of sex chromosomes in fungi, plants, and animals including insects, fish, and mammals [18], [56]–[58].

## Results

### Discovery of the sexual cycle of *C. heveanensis*

To study mating behavior and the *MAT* locus of *C. heveanensis*, the isolates (Table 1) were first analyzed to validate their species assignment. When the D1/D2 and ITS regions were sequenced and compared to the type strain CBS 569, all 9 strains were found to have identical D1/D2 sequences (Table 1, Figure S1). Seven isolates also had identical ITS sequences whereas two isolates (BCC 8396, BCC 11754) had one nucleotide difference in the ITS region (Table 1, Figure S1). These findings are in accord with the assignment of these isolates as *C. heveanensis*. Two other isolates (CBS 9459,CBS 6097) appear to represent related, but distinct species based on a higher level of divergence in the D1/D2 and ITS sequences (see Figure S1). All strains appear to be haploid based on fluorescence-activated cell sorting (FACS) analysis by comparison to *C. neoformans* haploid and diploid reference isolates (Table 1, Figure S2).

**Table 1:** Description of *C. heveanensis* isolates used in this study.

Strain number	Location of isolation	Substrate	Sequence difference compared to CBS 569		Ploidy based on FACS analysis	Self-filamentation on V8 medium (pH 5) in monoculture	Fertility	Mating type
			D1/D2	ITS				
CBS 569^T^	Indonesia	Sheet rubber	0	0	Haploid	**+**	fertile	*A1 B1*
BCC 8398	Thailand	Insect frass	0	0	Haploid	**+**	fertile	*A2 B2*
BCC 8396	Thailand	Insect frass	0	1	Haploid	**+**	fertile	*A1 B3*
BCC 11754	Thailand	Insect frass	0	1	Haploid	**+**	fertile	*A1 B3*
BCC 8384	Thailand	Insect frass	0	0	Haploid	**+**	sterile	*A1 B2* * §
BCC 8305	Thailand	Insect frass	0	0	Haploid	**-**	sterile	*A1 B4* *
BCC 8313	Thailand	Insect frass	0	0	Haploid	**-**	sterile	*A1 B4* *
BCC 11757	Thailand	Insect frass	0	0	Haploid	**-**	sterile	*A1 B5* *
BCC 15000	Thailand	Flower	0	0	Haploid	**-**	sterile	*A1 B6* *

**T**: type strain.

*: inferred mating type from molecular analysis.

§: the *B* locus of this strain was found to be very similar to BCC 8398 (92% for Sxi1 and 94% for Sxi2, with just one amino acid difference in the N-terminal dimerization region of Sxi1 and four amino acid differences in this region of Sxi2), but it could represent a different allele (B7).

**Figure 1:**
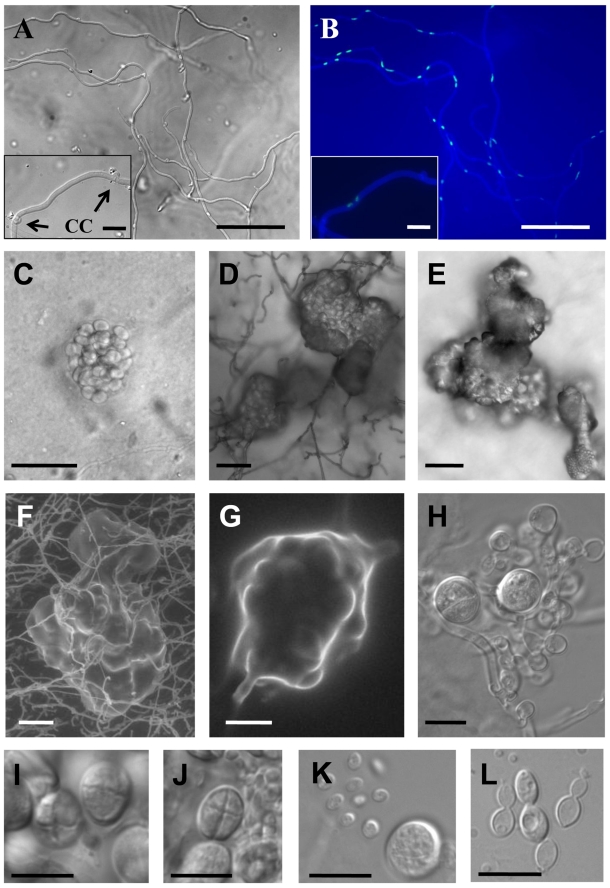
Sexual reproduction of *C. heveanensis*. (A,B) Hyphae with clamp connections. Scale bars represent 50 µm. Representative clamp connections (CC) are shown in the enlarged images located in the lower left panels (arrows, scale bars  = 10 µm). (A) Differential interference contrast (DIC) image, (B) fluorescence image showing cell walls, septa and nuclei stained with Calcofluor white and Sytox green. (C–E) DIC images of basidial clusters. Scale bars represent 50 µm. (C) A young basidial cluster embedded in the agar, (D,E) mature basidial clusters on the surface of the agar. (F,G) Scanning electron microscopy images of basidial clusters, scale bar in (F) is 50 µm, and in (G) is 10 µm. (H) A developing basidial cluster with two mature and numerous young basidia, scale bar  = 10 µm. (I and J) Globose basidia with cruciate septa. Scale bars represent 10 µm. (K) A basidium and basidiospores, scale bar  = 10 µm. (L) Vegetative yeast cells, scale bar  = 10 µm.

To test if this species has an extant sexual cycle, isolates were co-cultured and examined morphologically for production of mating specific structures. Strains were inoculated on V8 (pH 5 or 7) or MS media alone and in every pair-wise combination and incubated at room temperature in the dark or light. Five strains were self-filamentous when incubated alone while the other strains were not (Table 1). The filaments produced during monoculture were highly branched, irregular in shape, lacked clamp connections, and monokaryotic (nuclei stained with Sytox green) (data not shown). In contrast, dikaryotic hyphae with clamp connections and basidia fruiting bodies indicative of sexual reproduction were produced by three mating combinations (BCC 8398 × CBS 569, BCC 8398 × BCC 8396, and BCC 8398 × BCC 11754). Optimal mating conditions were on V8 medium (pH 5) in the dark. By 10 days incubation, compatible mating pairs had begun to produce dikaryotic filaments with clamp connections (Figure 1A and 1B), and basidia embedded in the agar were first apparent at 2 to 3 weeks of incubation (Figure 1C), and later developed into clusters in a matrix reaching the agar surface and visible by eye (Figure 1D–1G). The basidia are globose with a diameter of 7.5–10.5 µm and cruciate septa, similar to the related species *K. mangroviensis* and *Tremella mesenterica* (Figure 1H–1K, [55]). Basidiospores (subglobose, diameter 2.3–3.3 µm) were associated with the basidial clusters (Figure 1J and 1K) and were clearly distinguishable from the larger elliptical budding yeast cells (3.5–4.9 µm×5.0–7.2 µm) (Figure 1L). Fecundity was associated with self-filamentation (4/5 self-filamentous strains were fertile; 4 non-filamentous isolates were sterile (Table 1)). These morphological observations define an extant sexual cycle for this species. The dikaryotic sexual state that formed when compatible pairs of *C. heveanensis* were co-cultured was named *Kwoniella heveanensis* because the basidial morphology of this species is quite similar to that of *K. mangroviensis* (for Standard and Latin descriptions, see Materials and methods). Both species are phylogenetically related in a sister species clade to the pathogenic *Cryptococcus* species complex [54].

### Characterization of the *C. heveanensis MAT* locus

**Figure 2:**
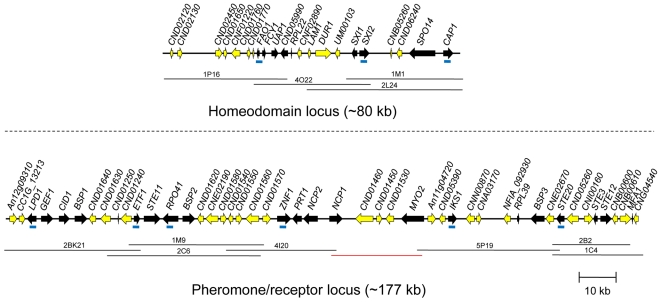
*C. heveanensis MAT* locus structure. *MAT* gene probes (represented by blue bars in the figure) were used to screen the fosmid library of *C. heveanensis* CBS 569. Sequencing the positive fosmids, represented by black lines, and subsequent assembly of the sequences generated two clusters corresponding to the homeodomain locus (∼80 kb) and the pheromone/receptor locus (∼180 kb). The genes that are present in the *MAT* locus of the pathogenic *Cryptococcus* species are shown in black, while others are shown in yellow. The red line indicates an ∼20 kb region sequenced by primer walking.

To elucidate the structure of the *C. heveanensis MAT* locus, probes to *MAT*-specific genes of *C. neoformans* and *C. gattii* were generated by degenerate PCR. These probes (*LPD1*, *RPO41*, *CAP1*, *ZNF1*, *STE20*, *IKS1*, *FAO1*, and *NOG2*) were used to screen a fosmid library for the type strain *C. heveanensis* CBS 569. Sequencing of positive fosmids and assembly of the sequences resulted in two large gene clusters: a region containing the homeodomain locus and flanking regions spanning ∼80 kb and a region containing the pheromone/receptor locus and flanking regions spanning ∼180 kb (Figure 2). The homeodomain locus of *C. heveanensis* contains two divergent homeodomain genes (*SXI1* and *SXI2*) encoding an HD1 and an HD2 class product, similar to other basidiomycete *MAT* loci [59]–[61]. Because the *MAT* locus alleles in *C. neoformans* and *C. gattii* contain only *SXI1*α or *SXI2* **a** but not both, this suggests that one or the other homeodomain gene has been lost recently in these pathogenic species.

**Figure 3:**
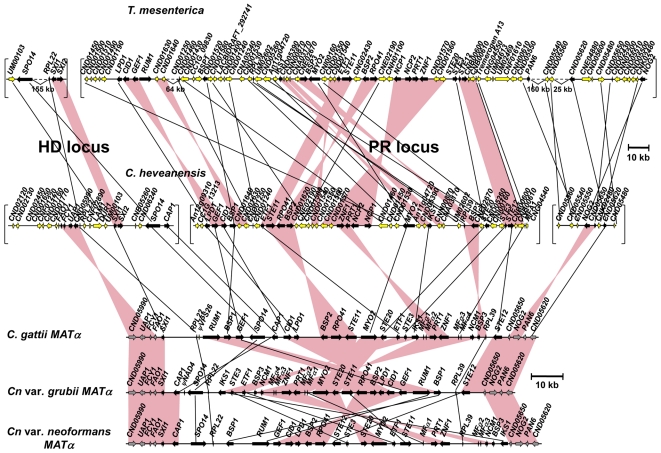
Synteny between *MAT* locus regions of *C. heveanensis*, *T. mesenterica*, and α alleles of *C. neoformans* var. *neoformans*, *C. neoformans* var. *grubii*, and *C. gattii*. C. heveanensis and *T. mesenterica* genes that are present in the *MAT* locus of *C. neoformans* and *C. gattii* are shown in black, while others are shown in yellow. Syntenic blocks containing more than one gene are shown as pink bars. The *T. mesenterica* and *C. heveanensis* homeodomain locus contains two homeodomain genes homologous with *SXI1* and *SXI2*; whereas, *C. neoformans* and *C. gattii* contain a single homeodomain gene, namely *SXI1* for α, and *SXI2* for the **a** allele. Upper scale bar refers to *T. mesenterica* and *C. heveanensis*, while lower scale bar refers to *C. neoformans* var. *neoformans*, *C. neoformans* var. *grubii* and *C. gattii*. JGI annotation IDs for *T. mesenterica* genes are listed in Table S1. The relative order and the orientations of the *C. heveanensis* and *T. mesenterica* contigs are unknown.

The genomic region containing the homeodomain (HD) locus of *C. heveanensis* also contains the *FAO1*, *FCY1*, and *UAP1* genes, which form the left boundary of the pathogenic *Cryptococcus* species *MAT* locus (Figure 3). Also included are the *RPL22*, *SPO14*, and *CAP1* genes in addition to the homeodomain genes, which are part of the *MAT* locus in *C. neoformans* var. *neoformans*, *C. neoformans* var. *grubii*, and *C. gattii* [46], [47], [49]. These three genes are also linked to the homeodomain genes of *Tremella mesenterica* (Figure 3).

The remaining genes found in the *MAT* locus of pathogenic *Cryptococcus* species are present in the region containing the pheromone/receptor (PR) locus of *C. heveanensis* (Figure 3). These include the pheromone receptor gene, *STE3*, the pheromone gene *MFA1*, the pheromone sensing MAPK cascade components *STE11*, *STE20* and *STE12*, and other genes whose functions are either unknown, essential, or may not be related to mating (including *BSP1*, *ETF1*, *ZNF1*, *PRT1*, *NCP1*, *MYO2*, *IKS1*, *RPL39*, and *BSP3*). This region also contains the *LPD1*, *GEF1*, *CID1*, *RPO41*, and *BSP2* genes, which were hypothesized to be present at the boundaries of the ancestral sex determining regions in the proposed evolutionary model (*RPO41* and *BSP2* in the ancestral homeodomain locus and *LPD1*, *GEF1* and *CID1* in the ancestral pheromone receptor locus) and to have been entrapped in *MAT* when the HD and PR loci were brought together via a chromosomal translocation [47]. The presence of these genes in the PR locus region of both *C. heveanensis* and *T. mesenterica* implies that these genes were likely to have all been linked to the ancestral PR locus prior to fusion to a contiguous bipolar sex-determinant.

**Figure 4:**
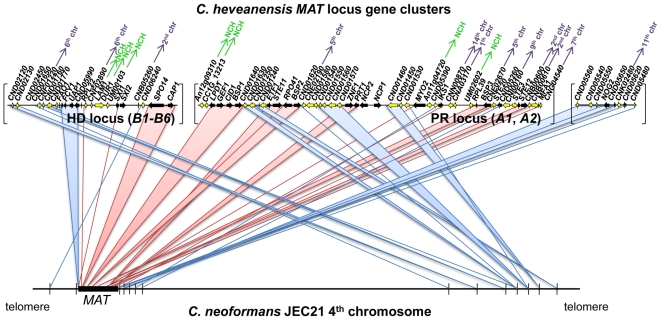
*C. heveanensis MAT* locus region shares synteny with the 4^th^ chromosome of *C. neoformans* JEC21. *C. heveanensis MAT* locus includes numerous genes that are not present in the *MAT* locus of *C. neoformans* or *C. gattii*. Although many of these genes are on the 4th chromosome of *C. neoformans* strain JEC21 where *MAT* is located, they correspond to the opposite telomeric region, suggesting intrachromosomal rearragements during the evolution of the *MAT* locus. Pink lines/bars depict the synteny between genes located within the *MAT* locus of *C. neoformans* and the corresponding genes in *C. heveanensis*. Blue lines/bars show the syntenic relationship of the genes that are linked to the *MAT* locus of *C. heveanensis*, but not part of the *MAT* locus of *C. neoformans*. The *C. heveanensis* genes whose *C. neoformans* homologs are located on different chromosomes are indicated with purple arrows with chromosome numbers. The genes that do not have apparent *C. neoformans* homologs are indicated by NCH, which stands for “no *C. neoformans* homolog”.

The HD and PR gene clusters of *C. heveanensis* have numerous other linked genes that are not present in the *MAT* locus of *C. neoformans* or *C. gattii*. Most of these genes (such as *CND01630*, *CND01640*, *CND05260*, *CND05390*, *CNN00870*, *CNE02670*, *CNI00160*, *CND01540*, *CNG02430*, *CNE02190*, *CND01240*, *CND01560*, and *CND01570*) are also present in the *T. mesenterica* PR locus region suggesting these genes have recently been lost from the *MAT* locus and relocated to other genomic locations in *C. neoformans* and *C. gattii* (Figure 4). Some of these genes do not have apparent homologs in *C. neoformans* or *C. gattii*, suggesting these genes may have been lost during the evolution of the *MAT* locus. It is also interesting that while a majority of these genes are on the 4^th^ chromosome of *C. neoformans* strains JEC21/B3501A, where *MAT* is also located, they correspond to the opposite telomeric region, suggesting intrachromosomal rearrangements may have occurred during *MAT* locus evolution (Figure 4, [62]).

### Homeodomain locus of *C. heveanensis* is multiallelic, and the *MAT*–specific region is likely to be restricted to the *SXI1* and *SXI2* genes

**Figure 5:**
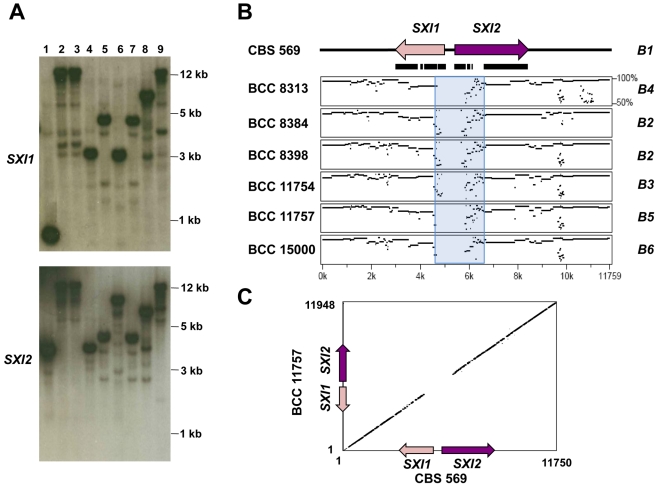
Homeodomain region of *C. heveanensis* is multiallelic and likely to be limited to the *SXI1* and *SXI2* genes. (A) Southern blot analysis showing hybridization patterns of *SXI1* and *SXI2* gene probes to EcoRV digested genomic DNA from *C. heveanensis* strains. Lane 1, CBS 569, 2, BCC 8305, 3, BCC 8313, 4, BCC 8384, 5, BCC 8396, 6, BCC 8398, 7, BCC 11754, 8, BCC 11757, 9, BCC 15000. (B) Percent sequence identity plots comparing an ∼12 kb region containing the *SXI1* and *SXI2* genes from CBS 569 with the corresponding region from other isolates. The blue box shows the highly dissimilar region corresponding to the N-terminal regions of Sxi1 and Sxi2. The black bars under the genes depict the exons. (C) A representative dot plot comparing the homeodomain region of CBS 569 (x-axis) and BCC 11757 (y-axis).

Southern blot analysis of the 8 additional *C. heveanensis* isolates and the type strain with *SXI1* and *SXI2* gene probes showed a highly divergent profile, suggesting the homeodomain locus structure of *C. heveanensis* might be multiallelic (Figure 5A). Strains BCC 8305 and BCC 8313 showed the same hybridization profile and sequence analysis also showed that the *SXI1*, *SXI2*, *LPD1*, *RPL22*, *ZNF1*, and *STE11* genes in these strains are 100% identical for the regions sequenced (data not shown). Strains BCC 8396 and BCC 11754 also appeared identical. Therefore, in these cases, one isolate from each strain pair was selected for further studies. Sequencing of an ∼12 kb region, spanning *SXI1* and *SXI2*, from seven strains provided evidence that the locus is multiallelic with 6 different alleles of *SXI1* and *SXI2* (Figure S3). Percent sequence identity plots comparing the homeodomain region of CBS 569 with the corresponding region from other isolates showed that there is a highly dissimilar core corresponding to the 5′ ends of the *SXI1* and *SXI2* genes and spanning a fairly restricted region of ∼2 kb (indicated by a blue box in Figure 5B) followed by a more similar region (∼80%), while the 3′ ends of the genes are almost identical. A representative dot plot comparing the homeodomain region of CBS 569 and BCC 11757 also shows the divergence at the 5′ ends of both genes (Figure 5C). Percent identity plots comparing the homeodomain region from each strain with the others are shown in Figure S4. The homeodomain genes of BCC 8384 and BCC 8398 were found to be very similar (Figure S3, Figure S4). When pairwise comparisons were conducted, percent identity of predicted amino acid sequences between BCC 8384 and BCC 8398 was found to be 92% for Sxi1 and 94% for Sxi2, while percent identity varied between 71–77% for Sxi1, 75–82% for Sxi2 for the other pairwise combinations. Considering the absence of mating between these two isolates, although they are both self-filamentous, these strains may have the same allelic versions of *SXI1* and *SXI2*, preventing mating.

**Figure 6:**
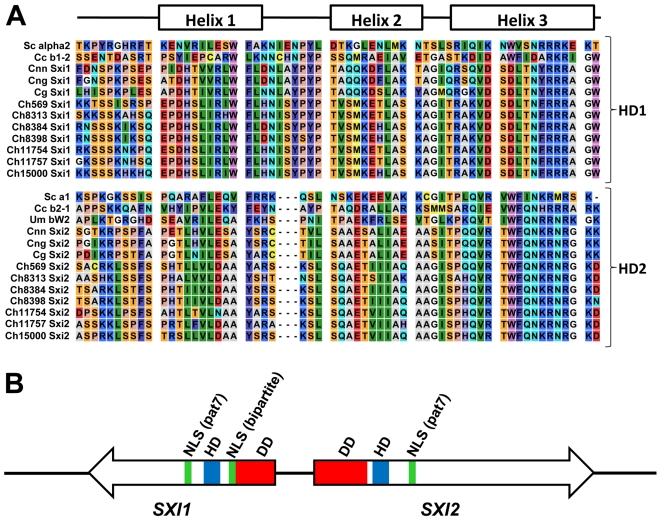
Sxi1 and Sxi2 from *C. heveanensis* align with HD1 and HD2 homeodomain proteins, respectively. (A) Alignment of the homeodomain regions of *S. cerevisiae* (Sc), *C. cinerea* (Cc), *C. neoformans* var. *neoformans* (Cnn), *C. neoformans* var. *grubii* (Cng), *C. gattii* (Cg), *C. heveanensis* (Ch) homeodomain proteins. These proteins are classified as either HD1 or HD2 according to the length of the homeodomain region. There are three extra amino acids between helix 1 and helix 2 of HD1 proteins. *C. heveanensis* Sxi1 and Sxi2 proteins are of type HD1 and HD2, respectively. (B) Schematic representation of the Sxi1 and Sxi2 domain structures. DD, HD, and NLS denote dimerization domain, homeodomain, and nuclear localization signal, respectively. The types of nuclear localization signal are indicated in parentheses: Bipartite and pat7, pat7 denotes the 7-residue pattern.

Homeodomain proteins have distinct domains for different functions [63]. The homeodomain is the DNA binding region consisting of 3 alpha helices. Fungal homeodomain proteins involved in mating are either HD1 or HD2 type according to the length of this homeodomain region [14]. In HD2 type homeodomain proteins, the distance between the first and second helix is three amino acids shorter than HD1 homeodomain proteins. The *C. heveanensis* Sxi1 and Sxi2 proteins are HD1 and HD2-type homeodomain proteins, respectively, like their *C. neoformans* counterparts (Figure 6A, [50], [51]).

Studies involving *S. cerevisiae* a1 and α2, *Coprinopsis cinerea* HD1 and HD2, and *U. maydis* bE and bW homeodomain proteins showed that following cell-cell fusion the two homeodomain proteins from compatible partners dimerize via their N-terminal domains [59], [64], [65]. These studies suggest that the interaction is through coiled-coils formed by alpha helices aligned such that the hydrophobic residues are adjacent. Although, we couldn't detect extensive coiled-coil formation in the *C. heveanensis* homeodomain proteins using the COILS software, there are 3–4 alpha helical regions in the N-terminal domains of both Sxi1 and Sxi2, which might form coiled-coil interactions (Figure S3).

In *C. cinerea*, two bipartite nuclear localization signals (NLSs) were found in HD1 and this region targeted a reporter protein to the nucleus [66]. In contrast, no NLS was apparent in the *C. cinerea* HD2 protein and HD2-reporter fusion proteins were not nuclear localized. Similarly in *U. maydis*, a bipartite NLS in bE (HD1) was predicted [67]. We found one bipartite and one 7-residue pattern (pat7) NLSs in Sxi1 (HD1), while only one pat7 type NLS in Sxi2 (HD2) using the PSORT II software (Figure 6B).

The *RPL22* and *CAP1* genes are part of the *MAT* locus in *C. neoformans* and *C. gattii* [47], [49]. The *RPL22* gene shares 88–90% identity among mating types while the 5′ and 3′ regions of the *CAP1* gene show different profiles. For *C. neoformans* var. *neoformans* and *C. neoformans* var. *grubii*, the identity is 90–92% for the 5′ region of *CAP1* and 84–85% for the 3′ region. For *C. gattii*, the identity is 88% for the 5′ region of *CAP1* and 93% for the 3′ region. Sequences from *RPL22* and *CAP1* were analyzed from *C. heveanensis* to determine if the HD locus encompasses these genes. When 530 bp of *RPL22* gene sequence from all 7 unique strains was analyzed, 97 to 99% sequence identity was observed. Similarly, comparison of 212 bp of *CAP1* gene sequence from strains CBS 569, BCC 8313, and BCC 15000 resulted in 99.5–100% identity. Based on this analysis revealing the *RPL22* and *CAP1* genes are more similar between *C. heveanensis* isolates than the **a** and α alleles of *C. neoformans*, it is likely that the HD locus of *C. hevenensis* only includes the homeodomain genes, *SXI1* and *SXI2*, although other linked genes should also be examined to further support this conclusion.

These results show that the HD locus of *C. heveanensis* is multiallelic with at least 6 different alleles determined among 7 strains. Thus, additional extant HD locus alleles likely remain to be discovered. In addition, the *MAT*-specific region is likely to be limited most prominently to the 5′ end of the *SXI1* and *SXI2* genes coding for the N-terminal regions of the proteins known to be involved in the specificity of heterodimer formation.

### Pheromone/receptor locus of *C. heveanensis* is at least biallelic

**Figure 7:**
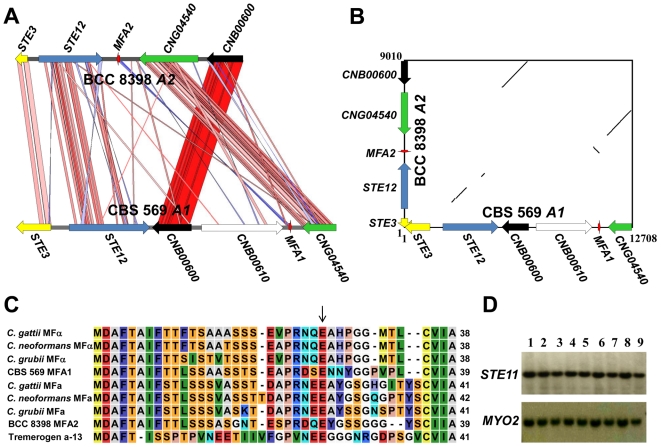
Analysis of the pheromone/receptor region of *C. heveanensis* isolates. (A) Comparison plot of the PR locus of CBS 569 and BCC 8398. The red and blue bands represent the forward and reverse matches, respectively. The genes are mostly in the same orientation except *MFA1/2*. The intensity of the color is proportional to the percent identity of the match, where red bands show regions of high sequence identity, while pink bands indicate lower sequence identity. (B) Dot plot analysis of the PR locus of CBS 569 and BCC 8398. (C) Sequence alignment of pheromone sequences from CBS 569, BCC 8398, *C. neoformans* var. *neoformans* MFα, *C. neoformans* var. *neoformans* MF**a**, *C. neoformans* var. *grubii* MFα, *C. neoformans* var. *grubii* MF**a**, *C. gattii* MFα, *C. gatii* MF**a** and *T. mesenterica* pheromone tremerogen a-13. The arrow indicates the N-terminal amino acid of tremerogen a-13 after proteolytic cleavage. (D) Southern blot analysis showing hybridization patterns of *STE11* and *MYO2* gene probes to EcoRV digested genomic DNA from *C. heveanensis* strains. Lane 1, CBS 569, 2, BCC 8305, 3, BCC 8313, 4, BCC 8384, 5, BCC 8396, 6, BCC 8398, 7, BCC 11754, 8, BCC 11757, 9, BCC 15000.

When the pheromone and the pheromone receptor gene sequences were amplified by PCR with CBS 569 specific primers, PCR fragments of the expected sizes were obtained for all strains except BCC 8398. Sequencing of these PCR products showed that the pheromone and receptor gene sequences were identical for all of the strains that yielded PCR products (data not shown). For the atypical strain BCC 8398, degenerate primers were used to amplify the *STE3*, *STE12*, *CNB00600*, and *CNG04540* genes and the gaps between these genes were closed by primer walking. This sequence analysis resulted in an ∼9 kb sequence spanning the 5′ end of *STE3*, *STE12*, a pheromone gene, which was named *MFA2*, and the *CNG04540* and *CNB00600* genes. All of these genes were divergent between BCC 8398 and CBS 569 except *CNB00600*. Sequence identity values are 54% for *STE3*, 53% for *STE12*, 75% for *MFA1/2*, and 63% for *CNG04540*. In addition, the gene order also differed as indicated in the comparison and the dot plot (Figure 7A and 7B). Interestingly, the divergent region between BCC 8398 and CBS 569 starts at the 3′ end of the *CNB00600* gene. The majority of this gene corresponding to the 5′ portion (1285 bases) is 98% identical, while for a small portion at the 3′ end (139 bases for CBS 569 and 124 bases for BCC 8398), the sequence identity is 53% suggesting this gene may have represented the border of *MAT* or have been subject to more recent gene conversion.

The pheromone genes of *C. heveanensis*, *MFA1* (from CBS 569) and *MFA2* (from BCC 8398), encode 39 amino acid-long pheromone precursors (Figure 7C). Both were identified by the characteristic C-terminal motif of lipopeptide pheromones: CAAX, where A is an aliphatic amino acid [68]. While Mfa1 ends with the motif CVIA like pheromone precursors from *C. neoformans*, *C. grubii*, *C. gattii* and *T. mesenterica*, Mfa2 has CIIA at its C-terminus. Tremerogen a-13, a *T. mesenterica* pheromone, was isolated and its structure determined [69]. According to this study, after proteolytic cleavage, the mature pheromone starts with a glutamic acid (E) residue indicated with an arrow in Figure 7C. Since this residue is conserved in all *Cryptococcus* pheromones, the proteolytic cleavage might occur at the same site in other pheromones as well.

No or minimal sequence differences were observed for the *LPD1* (100% sequence identity in 530 bases), *STE11* (99% in 470 bases), *ZNF1* (100% in 410 bases) or *IKS1* (99% in 1740 bases) genes. Also, Southern blot analysis using *STE11* and *MYO2* gene fragments as probes showed the same hybridization profile among all strains tested (Figure 7D). This shows that the divergent region is likely to be more restricted than that of *C. neoformans* and *C. gattii* and contains at least the receptor gene *STE3*, the pheromone gene *MFA1/2*, *STE12*, and *CNG04540*.

These results suggest that the PR locus of *C. heveanensis* is at least biallelic similar to *U. maydis* and *Tremella* species, and it contains not only *STE3* and *MFA1/2*, but also other genes including at least *STE12*, *CNB00600*, and *CNG04540*. Sequence analysis of the genomic regions flanking this region from the isolate BCC 8398 will be necessary to establish whether any additional regions of the PR *MAT* locus remain to be identified.

### Mating type genes exhibit three different phylogenetic patterns

**Figure 8:**
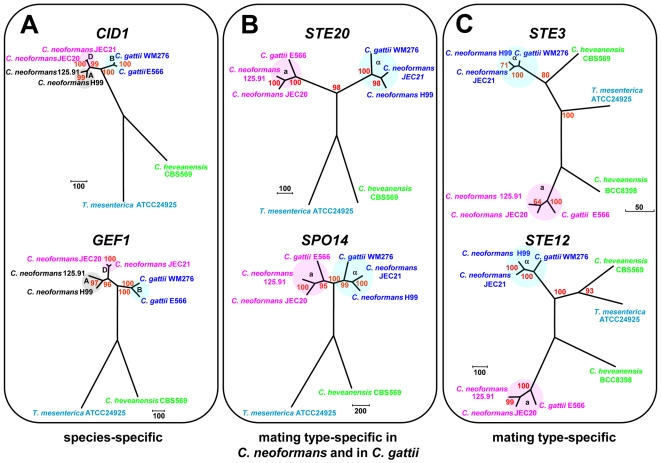
Mating type genes exhibit three phylogenetic patterns. (A) Species-specific profile exhibited by *CID1* and *GEF1*, (B) mating type-specific pattern exhibited by *STE20* and *SPO14* from *C. gattii* and *C. neoformans*, but not from *C. heveanensis* or *T. mesenterica*, (C) mating type-specific profile demonstrated by *STE3* and *STE12*.

Phylogenetic analyses were conducted on selected mating type genes (*CID1*, *GEF1*, *LPD1*, *BSP1*, *SPO14*, *ETF1*, *STE20*, *STE11*, *STE3* and *STE12*) from *C. heveanensis*, *T. mesenterica*, *C. neoformans* var. *neoformans*, *C. neoformans* var. *grubii* and *C. gattii* (Figure S5). These genes were classified into three groups based on their evolutionary history and two representative examples from each group are demonstrated in Figure 8. These analyses show that, *STE3* and *STE12* exhibit a mating type specific pattern, where *C. heveanensis* CBS 569 and *T. mesenterica* show a closer relationship to the α mating type group, while *C. heveanensis* BCC 8398 is closer to the **a** mating type group. *BSP1*, *SPO14*, *ETF1*, *STE20*, and *STE11* are mating type-specific in *C. neoformans* and *C. gattii*, however, none of these genes from either *C. heveanensis* or *T. mesenterica* followed a mating type-specific pattern. The third gene group consists of *CID1*, *GEF1* and *LPD1* that do not show a mating type-specific pattern, but demonstrate a species-specific pattern for all species.

## Discussion

We report here two advances with respect to understanding the evolution of sexual reproduction and mating type determination in fungi. First, we discovered and characterized the sexual cycle for *C. heveanensis*. Second, we cloned and sequenced the mating type locus for this organism. Unlike related species that commonly infect animals, *C. heveanensis* is not a pathogen and is instead associated with insects and flowers. This species represents an evolutionary window that provides novel insights into understanding transitions that occur between tetrapolar and bipolar mating type systems, and in model and pathogenic species. In particular, this analysis reveals how a tetrapolar ancestral species with two unlinked sex determining regions of the genome gave rise to bipolar extant species in which the sex determinants are linked, providing direct experimental validation of the central tenants of sex chromosome evolution originally proposed by Ohno [56]. These models involve the emergence of sex determinants on autosomes, which are then captured into a sex determining region or chromosome by sequential gene acquisition into strata of different evolutionary ages involving genes of coherent function. The basidiomycete mating type locus represents an exceptional experimental paradigm to test, and validate experimentally, key features of this model. That similar events have transpired repeatedly, and independently, in plants and animals and other fungi, illustrates common underlying principles that are involved in the emergence, evolution, and plasticity of genomic sex determining regions as diverse as fungal mating type loci and sex chromosomes.

Recent multilocus studies support the assignment of *C. heveanensis* to a clade of species related to but distinct from the pathogenic species *C. neoformans* and *C. gattii* [54]. Importantly, this analysis revealed that *C. heveanensis* occurs in a *sensu lato* group of species, including *K. mangroviensis*, which was recently discovered and found to have an extant sexual cycle that was defined as bipolar [55]. A critical aspect in finding the sexual cycle for *C. heveanensis* was the recent discovery of several new isolates for the species, which were made readily available by their deposition in a public strain collection [70]. Of three isolates previously reported to be *C. heveanensis*, our D1/D2 and ITS sequence analysis provides evidence that only the type strain CBS 569 is in fact *C. heveanensis*, whereas two other isolates (CBS 9459 and CBS 6097) appear to represent isolates of closely related cryptic species (Figure S1). Neither of these isolates was fertile with the *C. heveanensis* type strain. All eight of the recently reported isolates are, based on D1/D2 and ITS sequence analysis, bona fide *C. heveanensis* isolates. Pairwise incubation of all nine available *C. heveanensis* isolates under a broad set of conditions and media resulted in mating for three of the 36 possible mating combinations. Thus, at least four of the nine *C. heveanensis* isolates are fertile. Mating resulted in the production of dikaryotic hyphae with fused clamp connections, basidia with cruciate septa, and basidiospores. Notably, in solo incubations all four of the fertile strains were self-filamentous and produced monokaryotic hyphae, whereas only one of the sterile isolates was self-filamentous. Thus, the formation of monokaryotic hyphae by mating partners appears to be associated with and may precede successful mating, and these may facilitate location of mating partners or fusion of isolates. Based on the similarities of the sexual cycle between *C. heveanensis* and *K. mangroviensis*, we have assigned the teleomorphic designation *Kwoniella heveanensis*. In addition to providing insights into the evolution of mating and *MAT*, the discovery of the sexual cycle for *C. heveanensis* will also facilitate further laboratory investigations and strain construction.

How might the self-filamentous phenotype that may be necessary for sexual reproduction be controlled? Five of the nine *C. heveanensis* isolates are self-filamentous, and four of these are fertile in genetic crosses. The filaments produced may represent something similar to a conjugation tube, however as these occur during mono-culture, they are not induced by pheromones produced by a mating partner of compatible mating type. Possibilities include that elements of the pheromone response pathway have been constitutively activated by mutations, or in response to nutritional cues or limitation alone. Alternatively, it may be that these isolates are self-responsive to their own pheromone as a partial agonist of their Ste3 receptor homolog. In such a model, co-culture leads to more robust pheromone signaling necessary to enable cell-cell fusion and generation of a heterodimeric homeodomain complex that enables completion of sexual reproduction. Future studies to address these and other models will be necessary to test the activity and specificity of the mating pheromones and the Ste3 homologs involved in sensing them, and to isolate mutations that abrogate the self-filamentous phenotype testing if, for example, the pheromone and pheromone receptor genes are involved and whether this is necessary for mating.

**Figure 9:**
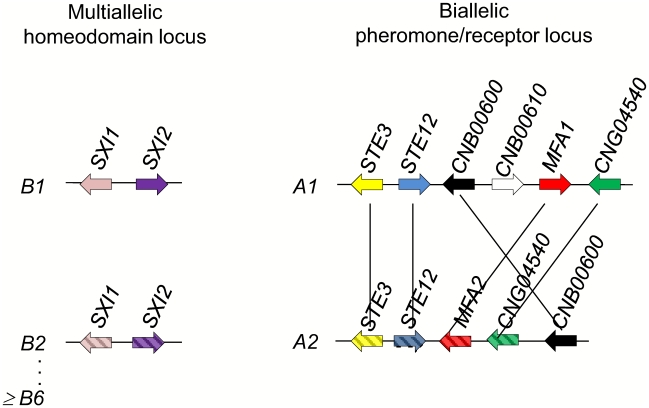
*C. heveanensis* is likely to have a tetrapolar mating type system with a multiallelic mating type locus and at least a biallelic pheromone/receptor locus. *C. heveanensis* has a multiallelic HD locus (*B* locus) including the genes *SXI1* and *SXI2* with at least 6 alleles among 7 unique strains examined. The PR locus (*A* locus) of *C. heveanensis* is at least biallelic and 6 of the strains have the *A1* allele, while one of the strains, BCC 8398, has the *A2* allele. The PR locus contains at least the *STE3*, *STE12*, *MFA1*/*2*, *CNB00600*, and *CNG04540* genes.

Analysis of the mating type locus for *C. heveanensis* revealed two apparently unlinked genomic regions, one corresponding to the homeodomain gene locus (*B*) and the second to the pheromone/pheromone receptor gene locus (*A*) (Figure 9). Notably, the *B* locus (HD) includes a pair of divergently oriented genes encoding homeodomain proteins related to Sxi1/Sxi2 from *C. neoformans* and bE/bW from *U. maydis*. The *A* locus (PR) includes genes encoding a Ste3 pheromone receptor homolog and a mating pheromone. Both loci are embedded in larger gene clusters, including some previously identified in the *C. neoformans MAT* locus and novel ones. To establish which genes in the ∼80 and ∼180 kb assemblies are mating type specific, representative regions were isolated by PCR and sequenced from fertile and sterile isolates. This analysis revealed striking diversity in the intergenic region and 5′ coding regions of the *SXI1/SXI2* genes, flanked by a less divergent region (∼80% identity) and followed by nearly identical 3′ coding regions (Figure 5). The divergent sequence region spans ∼2 kb. Importantly, this locus appears to be multi-allelic, based on sequence divergence, and at least 6 alleles are present in the 7 isolates examined, suggesting additional alleles may remain to be discovered. This *MAT* organization is quite similar to the *U. maydis b MAT* locus [59], [71], [72] corresponding to the diverse N-terminal coiled-coil dimerization domain that dictates self- vs. non-self recognition. The *RPL22* and *CAP1* genes linked to *SXI1/SXI2* in *C. heveanensis* had very similar sequences from the strains analyzed (97 to 100% identity), suggesting these genes are outside the mating-type specific region. Similar to other basidiomycetes, including *U. maydis*, *S. commune*, and *C. cinerea*, we suggest dimerization occurs between HD1 and an HD2 class homeodomain proteins encoded by different mating type locus alleles [65], [73], [74].

The *A* locus (PR) of *C. heveanensis* encodes a Ste3 pheromone receptor homolog, a mating pheromone, and other genes involved in pheromone sensing (Ste11, Ste12, and Ste20) are present or linked. Thus far, our sequence analysis for this large gene cluster spans ∼180 kb for the CBS 569 type strain and representative sequence from other isolates. This analysis allows us to conclude that the gene sequences, and the gene organization, differ substantially between two different alleles present in the population (Figure 7). The conclusion that at least two alleles are present is also supported by consideration of which strain combinations are fertile and which are infertile (Table 1). The finding that the *C. heveanensis A MAT* locus might be biallelic is similar to the tetrapolar species *U. maydis*, in which the *a* mating type locus is also biallelic [75]. The *C. heveanensis A* locus is distinguished from that of *U. maydis* in that additional genes are present within the locus, and the linked or flanking genes are also quite distinct. While further analysis of additional genomic regions from other isolates will be required to define the full extent of these *MAT* locus alleles, it is apparent that highly conserved sequences are embedded within more divergent ones (see Figure 7B). This may illustrate that gene conversion occurs within the locus, as is apparent in the *MAT* locus of *C. neoformans*, or that this *MAT* locus allele represents a transitional form into which related genes have been recruited but have not yet diverged into mating type specific allelic forms. Recent studies of the ectomycorrhizal fungus *Laccaria bicolor*, a tetrapolar species, have illustrated differences in the evolutionary trajectory for the two mating type loci [76]. Whereas the homeodomain locus appears to be in a region of the genome where syntenic gene organization is conserved, the pheromone and receptor gene locus is more dynamic and subject to different selective pressures, leading to dramatic changes in gene order and organization and insertion of many transposable elements. Similar properties may hold for the two mating type locus alleles of *C. heveanensis* and may be responsible for some of the unusual features of *MAT* in this and related species.

The molecular organization of the *C. heveanensis MAT* locus allows us to propose that this species has a tetrapolar mating type determination system. This conclusion is based on the finding of two apparently unlinked loci that correspond to the *A* and *B* mating type locus alleles of other tetrapolar fungal species [16], [77], [78]. Second, a high level of genetic sequence divergence is found for genes within each of the two loci, consistent with a role in mating type determination. Third, the *B* locus is multiallelic, as is the case of other tetrapolar fungi but not in bipolar species. Fourth, the specific alleles found for the isolates are well correlated with those that are in fact fertile in that all three successful combinations differ at both *MAT* loci, whereas for at least some of the infertile strain combinations, they share a closely related or identical allele at one or both *MAT* loci (see Table 1). Fifth, the finding that in the population that the *A1* locus allele occurs in isolates with at least six different highly diverged *B* locus alleles is consistent with two unlinked loci in linkage equilibrium defining mating type. Given the dramatic sequence divergence between the different *B* alleles, models in which these arose by divergence while linked to the *A1* locus seem less plausible. Put another way, if the *A* and *B* loci were to be linked, we would expect a higher proportion of the population would consist of *A1* linked to, for example, *B1*, than is observed.

The conclusion that *C. heveanensis* has a tetrapolar mating type determination system can be further tested by two approaches. First, additional physical evidence that the two *MAT* loci are unlinked in the genome, based on electrophoretic separation and demonstration by chromoblot that the two lie on different chromosomes, whole genome sequence analysis, or independent segregation following meiosis, would all provide evidence the two are unlinked. Thus far it has not been possible to resolve chromosomes of this species by pulsed field gel electrophoresis, even employing conditions that work effectively for the closely related species *C. neoformans*, *C. gattii*, and *C. amylolentus*. Whole genome analysis may prove to be a more facile approach. The evidence that the two *MAT* loci are unlinked is that we have been unable, thus far, to find fosmids that physically span both loci or provide evidence the two are linked. It is formally possible that the PR locus could be linked to the right most end of the HD locus, which would place the two ∼20 kb apart. We view this as unlikely as we found no evidence for fosmids in the library that would span such a theoretical region. It is formally possible that the two loci are on the same chromosome but far enough apart to be unlinked genetically, or that the two loci are partially linked. Future studies will be necessary to examine this in further detail.

Second, additional genetic evidence involving the isolation and analysis of meiotic progeny could further establish this species as tetrapolar. The sine qua non of a tetrapolar mating system is the production of four types of meiotic progeny (*A1 B1* crossed with *A2 B2* yielding four mating types: *A1 B1*, *A2 B2*, *A1 B2*, and *A2 B1*). Thus far we have not been successful in isolating basidiospores by micromanipulation, and this is a challenge as these are often embedded in a matrix together with basidia. Moreover, in our experience attempts to purify progeny from fungal mating mixtures often leads to a high rate of contamination from the parental isolates, confounding analysis. Future studies could approach this using genetically marked strains to enable selection of meiotic progeny to determine their mating type. A further implication of our findings and conclusions is that the mating type determination system for the closely related species *K. mangroviensis*, currently classified as a bipolar species with α and A mating types, may also turn out to be tetrapolar with further detailed analysis.

**Figure 10:**
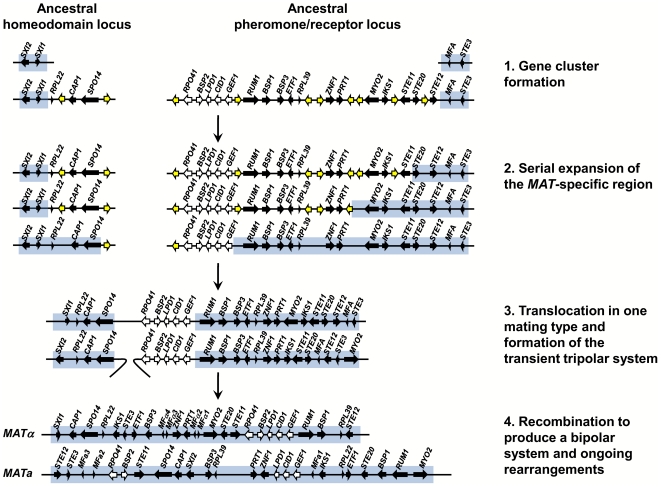
A model depicting the evolution of the bipolar *MAT* locus in the pathogenic *Cryptococcus* species from ancestral unlinked homeodomain and pheromone/receptor loci. First, mating-related genes together with other genes were acquired into two unlinked loci. Second, the mating type specific region including only the homeodomain genes in the HD locus and the pheromone and the pheromone receptor gene in the PR locus expanded serially by rearrangements or insertions leading to suppressed recombination. During expansion, some of the genes were relocated to different parts of the genome and others were lost. Third, the two loci were combined to a single locus in one mating type by a translocation event forming a transient tripolar system. Fourth, recombination between the mating types occurred to form the bipolar system. Ongoing inversions and rearrangements fashioned the extant *C. neoformans*/*C. gattii MAT* locus. Genes shown with black or white arrows are those found in the *C. neoformans*/*C. gattii MAT* locus, those in white exhibit a species-specific phylogeny, and those in yellow are not linked to the extant *MAT* locus in the pathogenic *Cryptococcus* species complex.

Our findings provide insight and direct experimental support for key features of an evolutionary model that was proposed earlier for the origins of the unusual bipolar mating type locus of the pathogenic *Cryptococcus* species complex based on a comparative genomic analysis of three closely related species (Figure 10) [47]. The proposed model hypothesized that the ancestral state was a tetrapolar organism with two unlinked loci, one encoding homeodomain genes and the other pheromones and the pheromone receptor that evolved in a stepwise fashion to the large bipolar mating type alleles in *C. neoformans* and *C. gattii*. In this model, two unlinked *MAT* loci first expanded by acquiring additional genes related to sexual reproduction, resulting in a series of gene strata of different evolutionary ages. Second, the two *MAT* loci fused via a translocation event in one of the two mating types, resulting in an intermediate “tripolar” state. Third, gene conversion between the linked and unlinked sex determinants resulted in fusion of the two loci in the other mating type and collapse to a bipolar state. Finally, more recent gene conversions and rearrangements occurred giving rise to the extant *MAT* alleles observed today.

Our analysis of the *C. heveanensis* mating type locus alleles reveals two large gene clusters, one corresponding to the left half of the *Cryptococcus* mating type locus encoding homeodomain genes and the other to the right half encoding pheromones, pheromone receptors, and signaling components. This organization serves as a molecular window on the organization of the *MAT* locus in the last shared common ancestor to the two lineages and represents key features of the hypothesized intermediate stages in the *MAT* evolutionary model (Figure 10). First, we see that the two ancestral *MAT* loci are probably unlinked and in some cases appear to have acquired additional related flanking genes. Second, the two large gene clusters have been formed, but have not yet undergone fusion to the tripolar or bipolar state. Given that two related large unlinked gene clusters are also present in the related species *C. amylolentus* (Findley and Heitman, unpublished results), the most parsimonious model is that the organization of *MAT* in *C. heveanensis* represents the ancestral state prior to fusion, rather than the result of a recent translocation that separated a contiguous *MAT* allele. Third, the strata of most recently acquired genes (*RPO41*, *BSP2*, *GEF1*, *CID1*, and *LPD1*) are all present in the right gene cluster linked to the pheromone and pheromone receptor genes, and thus were already present prior to linkage of the two regions. This differs in detail from the originally proposed model in which *RPO41* and *BSP2* had been depicted as linked to the homeodomain gene cluster, and *GEF1*, *CID1*, and *LPD1* as linked to the pheromone/receptor locus [47]. It also raises the likelihood that these genes are linked because they are functionally related in some way to *MAT* function, rather than being simply entrapped when the two unlinked *MAT* loci were fused by translocation. We note that the organization of the two gene clusters in *C. heveanensis* represents a juxtapositioning of *MAT*-specific genes (*SXI1*, *SXI2*) with genes that are part of the *MAT* locus in *C. neoformans*/*C. gattii* but not yet *MAT*-specific in *C. heveanensis*. Similarly, for the pheromone/receptor gene cluster, sequences that are not mating type-specific are embedded between mating type-specific sequences. These gene clusters therefore represent transitional intermediates representing *MAT* loci to which genes of related function have been recruited, but not yet captured into the mating type-specific regions of the genome or begun to diverge into mating type-specific alleles.

The model presented in Figure 10 represents our model for the events that led to the formation of a large bipolar mating type locus from a tetrapolar ancestral system. The proposed expansion of the ancestral HD and PR loci would likely have involved rearrangements facilitated by repetitive sequences that accumulate in recombinationally suppressed regions of the genome. If these genes have a function in sexual reproduction, as many do, this could have sustained the organization of the locus until the two loci fused. Some features of the gene strata apparent in the extant *C. neoformans* and *C. gattii MAT* alleles reflect episodic gene conversion events that occurred after the two ancestral *MAT* loci fused [44], [47]. Thus, multiple mechanisms operated involving acquisition of linked genes of related function into two expanding gene clusters, as is apparent here for the *STE12* gene linked to the PR locus, and gene conversion events between alleles both prior to and following linkage of the HD and PR loci that influenced the evolutionary trajectory and plasticity of this dynamic region of the genome.

A key finding of this comparative analysis is that two divergently oriented homeodomain genes are present in the *MAT* locus of *C. heveanensis*, whereas only one or the other homeodomain gene is present in the **a** or α *MAT* alleles of *C. neoformans* and *C. gattii*. This provides evidence that, like other tetrapolar basidiomycete fungi such as *U. maydis*, *Schizophyllum commune*, and *C. cinerea*, the ancestral Cryptococcal *MAT* locus contained two paired, divergent homeodomain genes and one or the other was lost recently in the *C. neoformans*/*C. gattii* lineage. Examples of homeodomain gene loss are also seen in *MAT* of pathogenic *Candida* species [79]–[81]. For example, the alpha2 homeodomain gene has been lost in two haploid emerging yeast pathogenic species, *Candida lusitaniae* and *Candida guillieromondii*, both of which have retained complete sexual cycles, and in addition *C. guilliermondii* has also lost the a1 homeodomain gene. As discussed elsewhere [17], the transition from paired to solo homeodomain genes further serves to restrict outbreeding potential. In species with paired homeodomain genes, there are two opportunities to form functional heterodimeric pairs following cell fusion, whereas species with only a single *MAT* encoded homeodomain protein have only one chance to form a productive heterodimer required for completion of sexual reproduction. A second key finding is that the *C. heveanensis A* locus (PR) is at least biallelic; when additional isolates become available it will be possible to test if other extant alleles are present (triallelic or multiallelic). Previous studies of *U. maydis*, which is tetrapolar with a biallelic pheromone/pheromone receptor locus [75], and the closely related species *Sporisorium reilianum* [77], which is tetrapolar and triallelic at the pheromone/pheromone receptor locus, provide evidence that transitions have occurred in other fungi from multiallelic/multiallelic to multiallelic/biallelic mating type determination systems. These transitions involving the loss of allelic diversity at one of the two unlinked mating type alleles result in a relative decrease in outcrossing frequency but do not alter the restriction to inbreeding engendered by the tetrapolar state [17]. Thus, a loss of allelic diversity at the *A* locus encoding pheromone and pheromone receptors may have similarly occurred in the lineages leading to *C. heveanensis*. The outcome for the species then in both cases is a restriction in outbreeding potential, which may serve to further promote inbreeding and clonality in the population, and this transition in sexuality may have contributed to the emergence of this clade of organisms as successful pathogens of animals, including humans.

## Materials and Methods

### Strains and media

Strains used in this study are listed in Table 1. *C. heveanensis* type strain CBS 569, CBS 9459, and CBS 6097 were obtained from the Centraalbureau voor Schimmelcultures (CBS), Netherlands. Other strains of *C. heveanensis* were from the BIOTEC Culture Collection (BCC), Thailand [70]. Strains were grown in YPD broth or on YPD solid medium. Mating assays were performed on V8 medium (pH 5 and pH 7) [82] and Murashige and Skoog (MS) medium minus sucrose (Sigma-Aldrich, [83]) by pairwise inoculation of strains and incubation at room temperature in the dark on dry upright media without parafilm.

### Standard and Latin descriptions of the sexual stage

Standard description: *Kwoniella heveanensis* Metin, Findley, & Heitman sp. nov.

Etymol.: The epithet is chosen to be identical with that of *Cryptococcus heveanensis* (Groenewege) Baptist & Kurtzman comb. nov [84].

Heterothallic fungus. Hyphae dikaryotic, clamp connections fused. Basidia clustered, submerged initially, finally on aerial hyphae, globose, 7.5–10.5 µm, cruciately septate, sterigmata not observed. Basidiospores subglobose, 2.3–3.3 µm, germinating by conidia.

Latin description: *Kwoniella heveanensis* Metin, Findley, & Heitman sp. nov.

Species heterothallica. Hyphae dikaryoticae, fibulae fusae. Basidia aggregata, primum submersa, deinde superficialia, globosa, 7.5–10.5 µm diam, septis cruciatis divisa, sterigmatibus carentia, Basidiosporae subglobosae, 2.3–3.3 µm diam, cito conidia proferentia.

### Fluorescence-activated cell-sorting (FACS) analysis

The protocol for the preparation of cells for FACS was based on a previous protocol [85]. Briefly, ∼50 µl of cells scraped from overnight grown cultures of YPD plates were washed with PBS and fixed in 1 ml of 70% ethanol overnight. Then the tubes were centrifuged and the supernatants were discarded. After washing the cells with 1 ml of NS buffer (10 mM Tris-HCl pH 7.2, 0.25 M sucrose, 1 mM EDTA, 1 mM MgCl_2_, 0.1 mM CaCl_2_, 0.1 mM ZnCl_2_), they were resuspended in 180 µl NS buffer. Then, 20 µl RNase A (10 mg/ml, Qiagen) and 5 µl of propidium iodide (0.5 mg/ml, CALBIOCHEM) were added. After incubating at room temperature in the dark with mixing for at least 2 hours, 17.5 µl of the cells were mixed with 700 µl of 50 mM Tris-Cl pH 7.5 and the tubes were sonicated for 45 seconds. Flow cytometry was performed on 10,000 cells and analyzed on the FL1 channel with a Becton-Dickinson FACScan.

### Microscopy

For microscopic examination of mating, either the mating mix was inoculated into V8 medium (pH 5) medium plates, and later a block of agar containing the hyphae and basidia was transferred to microscope slides, or the mating mix was directly inoculated onto slides containing a thin layer of mating medium.

For staining, a modified version of a previously published protocol was used [86]. Calcofluor white (0.05% solution, BD) and Sytox green (5 mM, Invitrogen) were used to stain the cell wall and nuclei, respectively. The slide containing the agar piece with hyphae or basidia was first washed with PBS, then, 5–10 µl of Calcofluor white was added and mixed gently with a cover slip. After incubating for 15 min, the excess dye was washed away with PBS. Then fixing was performed by incubating the slide in fixing solution (3.7% formaldehyde, 1% triton-X100 in PBS) for 15 min and the slide was washed with PBS. After that, 5 to 10 µl of 1∶10 diluted Sytox green was spotted on the slide, mixed with a cover slip, and incubated for 15 min. Then the slide was washed with PBS, mounted with a cover slip, and examined under the microscope. Microscopy was performed with an Axioskop 2 plus upright microscope (Zeiss). Images were captured using an AxioCam MRm camera and AxioVision application software (Zeiss). Scanning electron microscopy was performed with an XL30 environmental SEM (FEI).

### Fosmid library preparation

For library production, the CopyControl Fosmid Library Production Kit (Epicentre) was used according to the manufacturer's instructions. In each library, a total of ∼16,000 clones were picked into 96 well plates. Purified genomic DNA (∼40 kb fragments) was first sheared, end-repaired, and separated using contour-clamped homogenous electric field (CHEF) on a CHEF DR-II apparatus (Bio-Rad). The following conditions were used: 1- to 6-sec switch time, 6 V/cm, 14°C for 14 to 15 hrs. The size-fractionated DNA was recovered by gel extraction and precipitated. The insert DNA was then ligated into the CopyControl pCC1FOS cloning-ready vector and incubated overnight at room temperature. The ligated DNA was packaged and plated on *E. coli* phage-resistant cells overnight at 37°C. Fosmid clones were then picked into 96 well plates and eventually transferred to 384 well plates, which were stored at −80°C.

### DNA extraction, degenerate PCR, and library screening

For DNA isolation, strains were inoculated in 50 ml YPD medium, grown for two days at room temperature with shaking and centrifuged. After freezing at −80°C, the pellet was freeze-dried overnight and DNA was isolated as previously described [87].

Degenerate primers were designed for *LPD1*, *RPO41*, *CAP1*, *ZNF1*, *STE20*, *IKS1, FAO1* and *NOG2* based on known gene sequences from *C. neoformans* var. *neoformans*, *C. neoformans* var. *grubii*, and *C. gattii*. These primers (Table S2) were used in PCR reactions with *C. heveanensis* genomic DNA using Ex Taq polymerase (Takara) and the products obtained were gel extracted with QIAquick Gel Extraction Kit (Qiagen). The PCR fragments were cloned into pCR 2.1-TOPO using the TOPO TA Cloning Kit (Invitrogen). From the resulting colonies, plasmid isolation was performed with QIAprep Spin Miniprep Kit (Qiagen) and the plasmids were sequenced with M13F and M13R vector specific primers. After obtaining *MAT* gene sequences, specific primers were designed using a web-based program, Primer3 (http://frodo.wi.mit.edu/). After PCR with specific primers, the resulting gene fragments were gel-extracted as previously described and used as probes to screen the library.

For library screening, colony lifts were performed on Hybond membranes (Amersham) using standard protocols [88]. The PCR products were labeled with Prime-It II Random Primer Labeling Kit (Stratagene) with dCTP. Hybridization was performed using ULTRAhyb hybridization buffer (Ambion) according to the manufacturer's instructions.

### Sequencing and assembly

Positive fosmids were isolated from 200 ml of overnight grown cultures using the Qiafilter plasmid midi kit (Qiagen). The fosmid DNA obtained was sheared using a hydroshear apparatus (Genomic Solutions) to 1.6–3 kb fragments. The gel extracted sheared DNA was then ligated to adapters. To prepare the vector, pUC18 plasmid DNA was cut with EcoRI and HindIII removing the polylinker site, and ligated to single stranded oligos that contain one end complementary to the restriction enzyme site, and the other non-complementary to itself to prevent self-ligation. The sheared and adapter-ligated DNA was then ligated to the pUC18-derived vector and the reaction was used to transform DH5α competent cells (Invitrogen). White colonies were inoculated onto 96 well plates and plasmid isolation was performed with DirectPrep 96 MiniPrep kit (Qiagen). For each positive fosmid, four to five 96 well plates were used.

Sequencing reactions were performed with BigDye Terminator v3.1 Cycle Sequencing Kit (Applied Biosystems) and analyzed on PE3700 96-capillary sequencer (Applied Biosystems) in the Sequencing Facility at Duke University. The sequence reads were assembled using Phred, Phrap, and Consed program packages [89]–[91]. The gaps were closed by designing primers from contig ends using Primer3, amplifying across gaps by PCR and sequencing both strands. Short gaps were closed using iProof polymerase (Biorad) and long gaps were closed using LA *Taq* polymerase (Takara).

### Southern blot analysis

For Southern hybridization of *C. heveanensis* strains, 5 µg of genomic DNA from each strain was digested with EcoRV, electrophoresed on a 0.8% agarose gel, and blotted onto Hybond membranes (Amersham) using standard protocols [88]. The membrane was then hybridized to *MAT* gene probes generated by PCR as described above.

### Annotations and bioinformatic analyses

Annotations of the genes were done manually by comparing the 3-frame translated *C. heveanensis* sequences with the most similar protein sequences identified by BLASTX. Genbank accession numbers are, GU129941 for CBS 569 homeodomain locus, GU205379 for CBS 569 pheromone/receptor locus, GU129940 for BCC 8398 pheromone/receptor locus, GU129942, GU129943, GU129944, GU129945, GU129946, and GU129947 for homeodomain loci of the strains BCC 8313, BCC 8384, BCC 8398, BCC 11754, BCC 11757, AND BCC 15000, respectively. Genbank accession numbers for D1/D2 and ITS regions are GU585738 and GU585749 for CBS 9459, GU585739 and GU585750 for CBS 6097, GU585740 and GU585751 for BCC 8305, GU585741 and GU585752 for BCC 8313, GU585742 and GU585753 for BCC 8384, GU585743 and GU585754 for BCC 8396, GU585744 and GU585755 for BCC 8398, GU585745 and GU585756 for BCC 11754, GU585746 and GU585757 for BCC 11757, and GU585747 and GU585758 for BCC 15000, respectively. The accession number of the ITS region of CBS 569 is GU585748.

Percent sequence identity plots and dot plots were generated using the MultiPipMaker [92]. Pairwise sequence identity values were calculated using Geneious 4.6.4.

The comparison plot between pheromone/receptor loci of strains CBS 569 and BCC 8398 was produced using Artemis Comparison Tool Release 8 [93]. The input comparison file was generated using WebACT with the tBlastx algorithm.

Homeodomain regions of the proteins Sxi1 and Sxi2 were predicted by comparison to the previously characterized homedomain proteins. Helical content of the N-terminal domains of Sxi1 and Sxi2 were predicted with Jpred 3 [94]. Nuclear localization signals were predicted with PSORT II [95].

### Phylogenetic analysis

Phylogenetic analysis was conducted on coding sequences using MEGA4 [96]. The phylogenetic relationship was inferred using the Maximum Parsimony method.

## Supporting Information

Figure S1 Eight BCC isolates are confirmed to be *C. heveanensis*, while the strains CBS 9459 and CBS 6097 are closely related but different species. Eight isolates obtained from BCC have identical D1/D2 sequence as the *C. heveanensis* type strain CBS 569, while there are two differences between CBS 569 and CBS 9459, and three differences between CBS 569 and CBS 6097. Similarly, six of the eight BCC strains have identical ITS sequence as the type strain CBS 569, and there is only one difference between the type strain and the strains BCC 8396 and BCC 11754. However, there are eleven differences between CBS 569 and CBS 9459, and eight differences between CBS 569 and CBS 6097 in the ITS region.
(0.82 MB PDF)

Figure S2 *C. heveanensis* isolates are haploid based on FACS analysis. The type strain CBS 569 and eight BCC isolates were found to be haploid based on FACS analysis conducted with the haploid control *C. neoformans* JEC20 and the diploid control *C. neoformans* XL442.
(0.24 MB PDF)

Figure S3 Sequence alignment of homeodomain proteins. (A) Sxi1, and (B) Sxi2 sequence alignments. Purple boxes indicate the alpha helical regions at the N-terminal region of the proteins. Blue box shows the homeodomain region and green boxes indicate the predicted nuclear localization signals.
(2.26 MB PDF)

Figure S4 Percent sequence identity plots of the homeodomain region. An extended version of Figure 5B including percent sequence identity plots comparing an ∼12 kb homeodomain region from (A) BCC 8313, (B) BCC 8384, (C) BCC 8398, (D) BCC 11754, (E) BCC 11757, (F) BCC 15000 with the corresponding region from other isolates. The aligned regions are shown in green and well aligned regions (at least 70% sequence identity) are shown in pink.
(0.12 MB PDF)

Figure S5 Phylogenetic analysis of selected mating type genes. An extended version of Figure 8. (A) Species-specific profile exhibited by *CID1*, *GEF1*, and *LPD1*, (B) mating type-specific pattern exhibited by *BSP1*, *SPO14*, *ETF1*, *STE20* and *STE11* from *C. gattii* and *C. neoformans*, but not from *C. heveanensis* or *T. mesenterica*, (C) mating type-specific profile demonstrated by *STE3* and *STE12*, and (D) *SXI1* and *SXI2* sequence alignments from *C. heveanensis* strains, *C. gattii* and *C. neoformans*.
(0.14 MB PDF)

Table S1 JGI annotation IDs for *T. mesenterica* genes presented in Figure 3.
(0.02 MB PDF)

Table S2 Primer sequences.
(0.06 MB XLS)
