# Supplementary material for: The Mating Type Locus (MAT) and Sexual Reproduction of Cryptococcus heveanensis: Insights into the Evolution of Sex and Sex-Determining Chromosomal Regions in Fungi
Source: PLoS Genet. 2010 May 20;6(5):e1000961. doi: 10.1371/journal.pgen.1000961 (PMC2873909; doi:10.1371/journal.pgen.1000961)
Supplement: Figure S2 — C. heveanensis isolates are haploid based on FACS analysis. The type strain CBS 569 and eight BCC isolates were found to be haploid based on FACS analysis conducted with the haploid control C. neoformans JEC20 and the diploid control C. neoformans XL442. (0.24 MB PDF) [file pgen.1000961.s002.pdf]

Figure S2

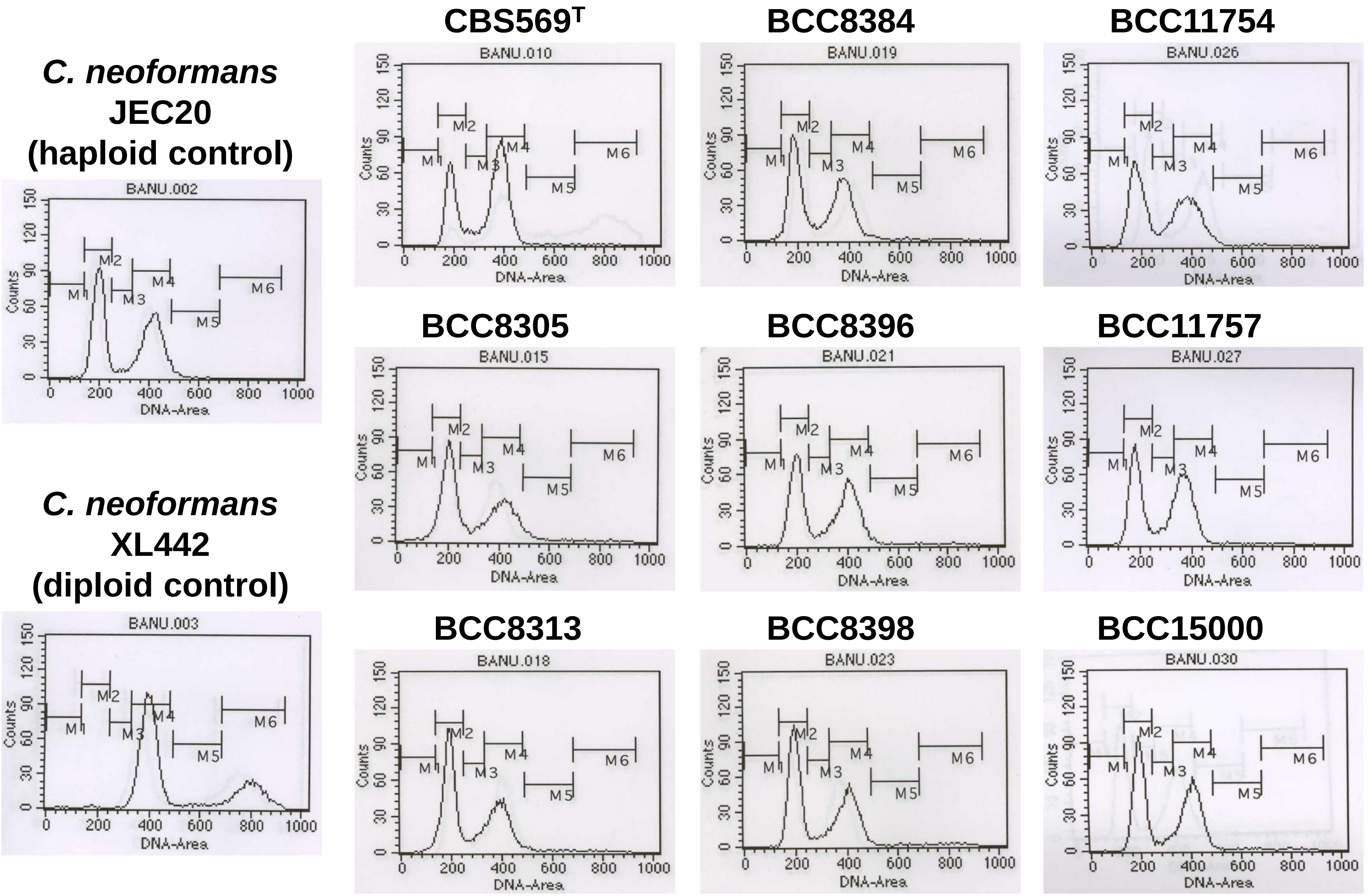

C. neoformans
JEC20
(haploid control)
BANU.002
C. neoformans
XL442
(diploid control)
BANU.003
CBS569T
BANU.010
BCC8384
BANU.019
BCC11754
BANU.026
BCC8305
BANU.015
BCC8396
BANU.021
BCC11757
BANU.027
BCC8313
BANU.018
BCC8398
BANU.023
BCC15000
BANU.030
Counts
DNA-Area
M1
M2
M3
M4
M5
M6
