# Supplementary figures and images for: The Mating Type Locus (MAT) and Sexual Reproduction of Cryptococcus heveanensis: Insights into the Evolution of Sex and Sex-Determining Chromosomal Regions in Fungi
Source: PLoS Genet. 2010 May 20;6(5):e1000961. doi: 10.1371/journal.pgen.1000961 (PMC2873909; doi:10.1371/journal.pgen.1000961)

Figure S3 (A)

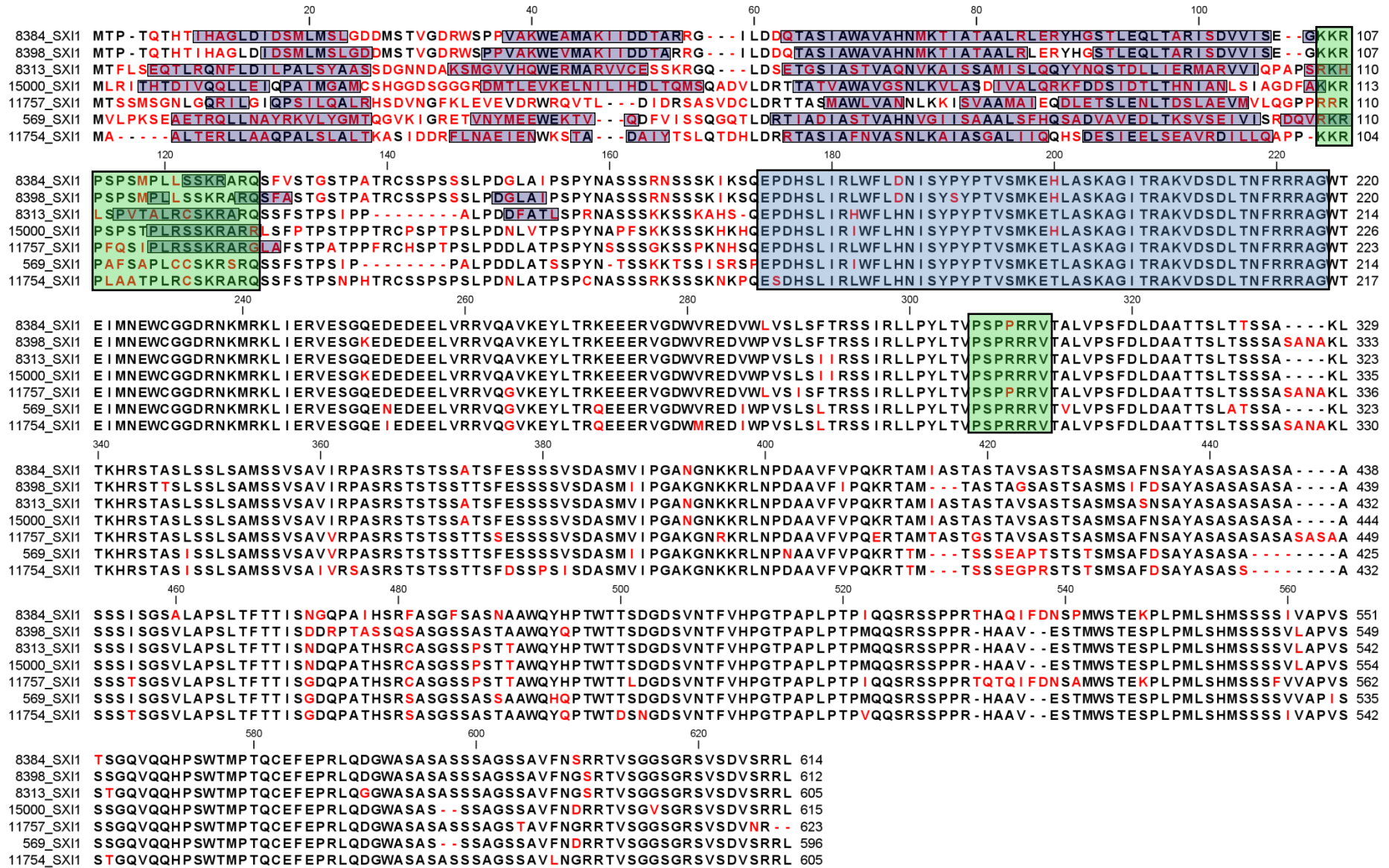

Figure S3 (B)

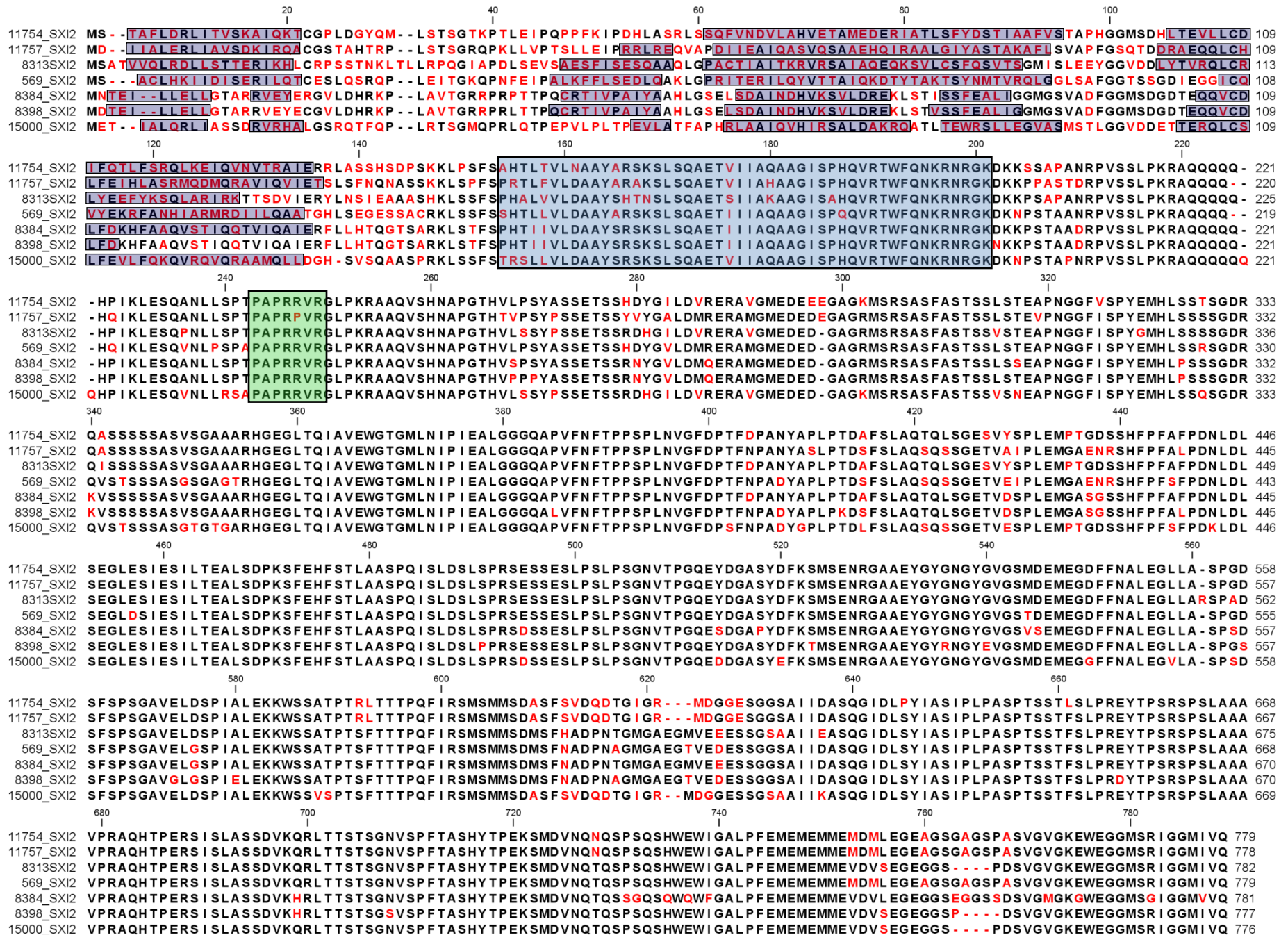

Supplement: Figure S3 — Sequence alignment of homeodomain proteins. (A) Sxi1, and (B) Sxi2 sequence alignments. Purple boxes indicate the alpha helical regions at the N-terminal region of the proteins. Blue box shows the homeodomain region and green boxes indicate the predicted nuclear localization signals. (2.26 MB PDF) [file pgen.1000961.s003.pdf]

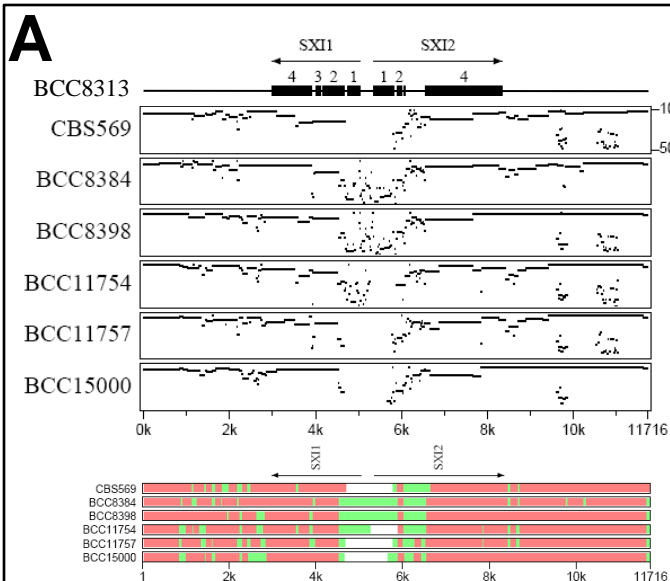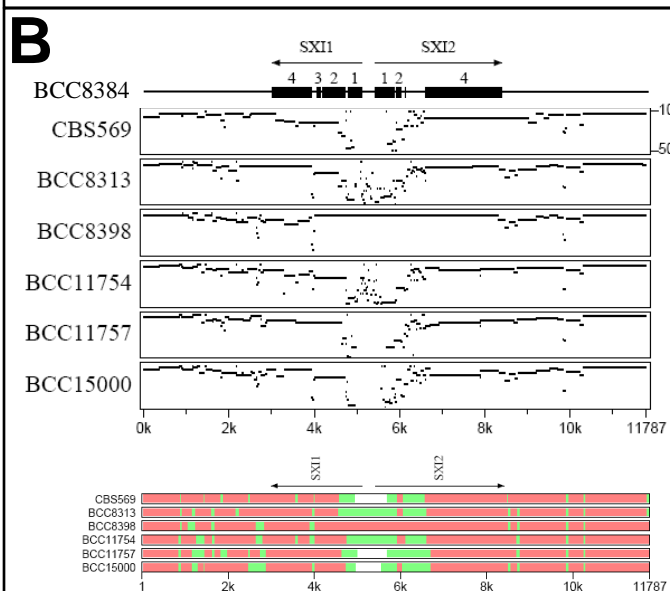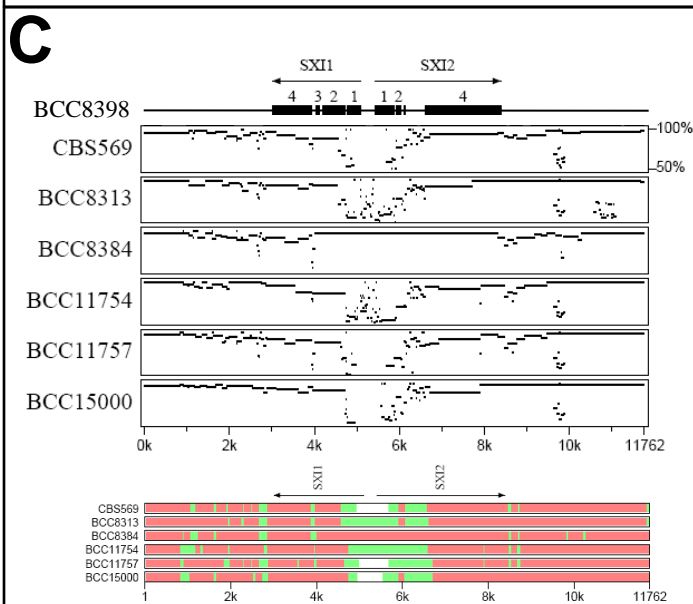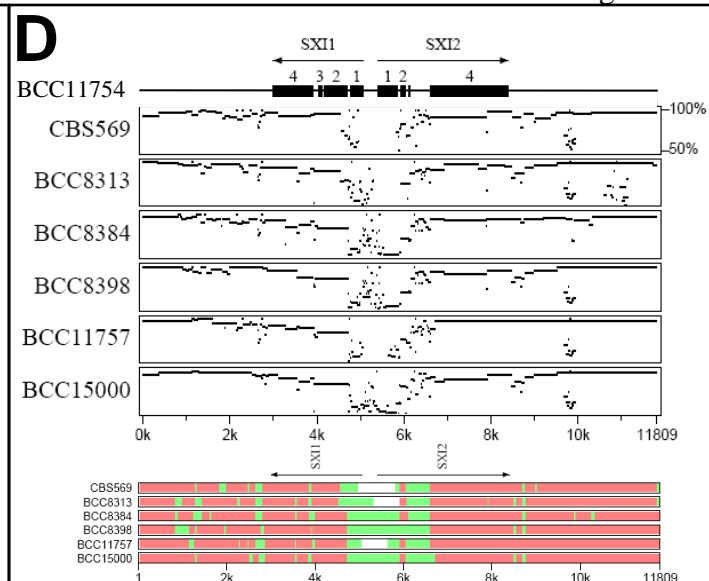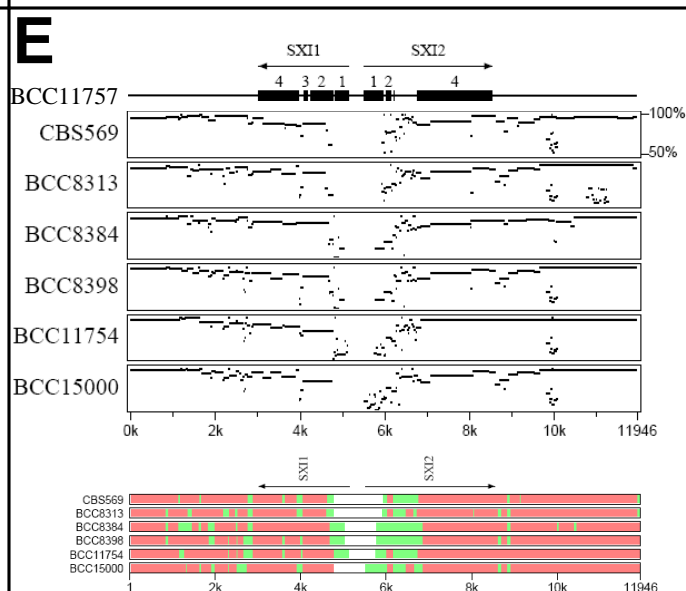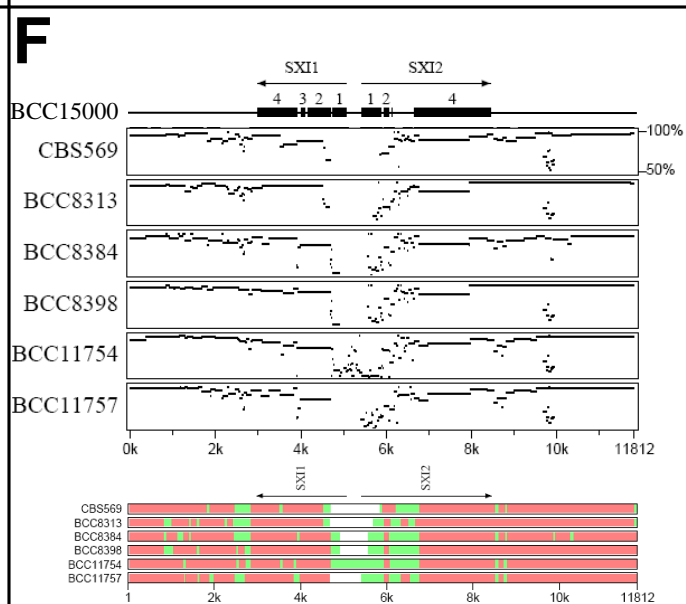

Supplement: Figure S4 — Percent sequence identity plots of the homeodomain region. An extended version of Figure 5B including percent sequence identity plots comparing an ∼12 kb homeodomain region from (A) BCC 8313, (B) BCC 8384, (C) BCC 8398, (D) BCC 11754, (E) BCC 11757, (F) BCC 15000 with the corresponding region from other isolates. The aligned regions are shown in green and well aligned regions (at least 70% sequence identity) are shown in pink. (0.12 MB PDF) [file pgen.1000961.s004.pdf]

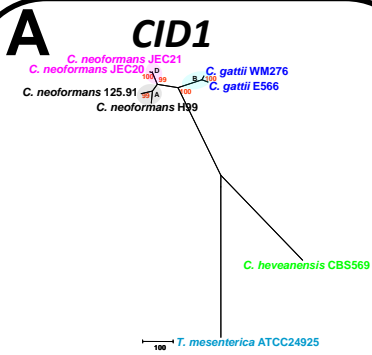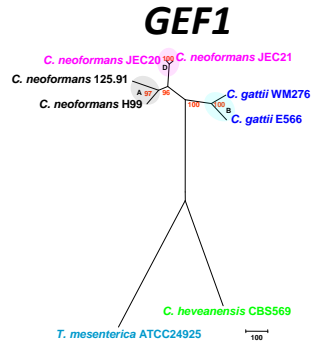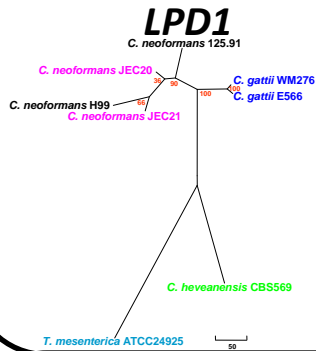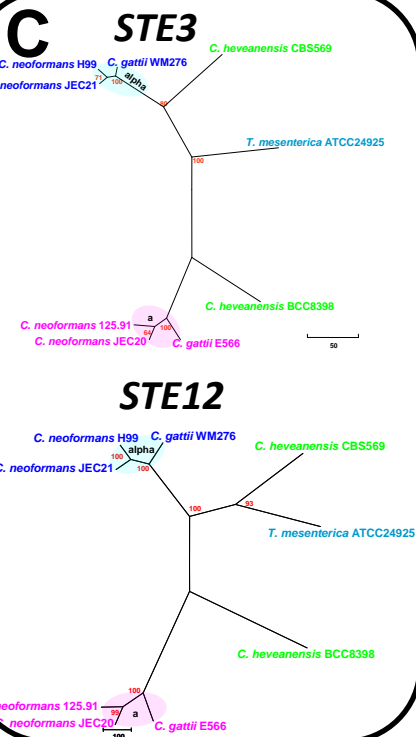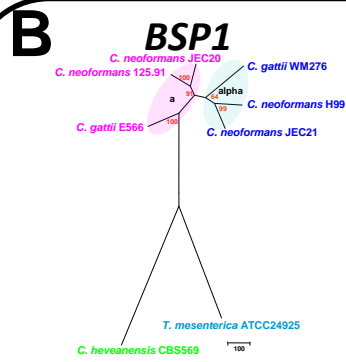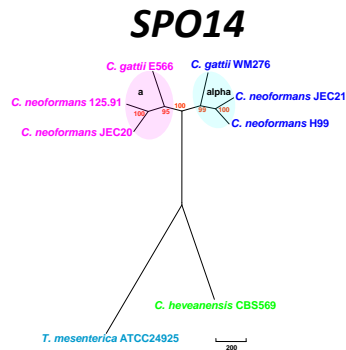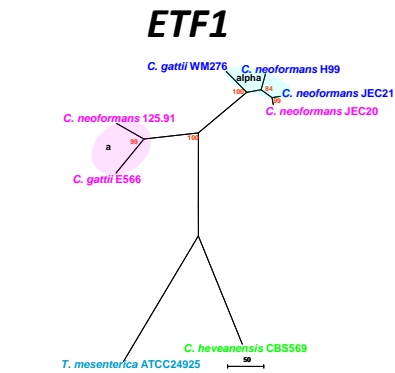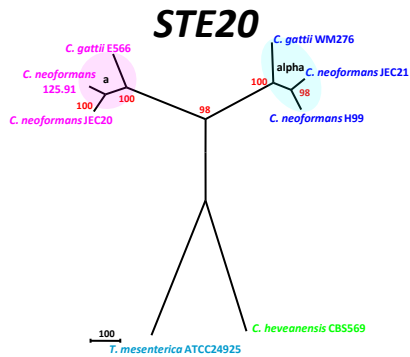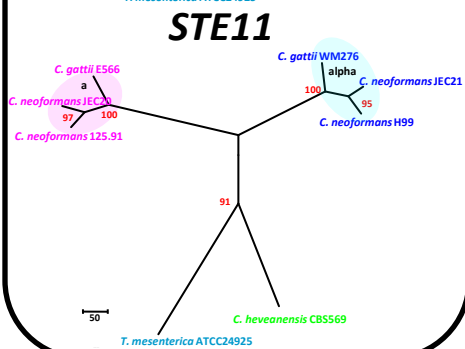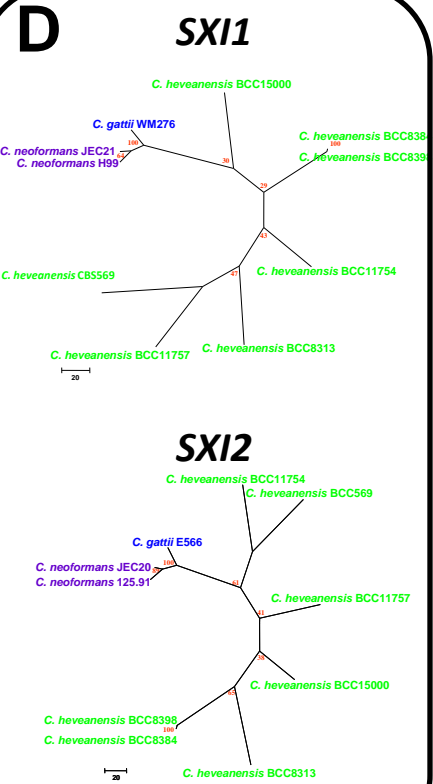

Supplement: Figure S5 — Phylogenetic analysis of selected mating type genes. An extended version of Figure 8. (A) Species-specific profile exhibited by CID1, GEF1, and LPD1, (B) mating type-specific pattern exhibited by BSP1, SPO14, ETF1, STE20 and STE11 from C. gattii and C. neoformans, but not from C. heveanensis or T. mesenterica, (C) mating type-specific profile demonstrated by STE3 and STE12, and (D) SXI1 and SXI2 sequence alignments from C. heveanensis strains, C. gattii and C. neoformans. (0.14 MB PDF) [file pgen.1000961.s005.pdf]
