# Supplementary material for: The Mating Type Locus (MAT) and Sexual Reproduction of Cryptococcus heveanensis: Insights into the Evolution of Sex and Sex-Determining Chromosomal Regions in Fungi
Source: PLoS Genet. 2010 May 20;6(5):e1000961. doi: 10.1371/journal.pgen.1000961 (PMC2873909; doi:10.1371/journal.pgen.1000961)
Supplement: Table S1 — JGI annotation IDs for T. mesenterica genes presented in Figure 3. (0.02 MB PDF) [file pgen.1000961.s006.pdf]

Table S1

T. mesenterica gene name	JGI Annotation ID
NOG2	31855
PAN6	32058
TREMEROGEN A13	-
STE12	31921
STE20	71885
STE3	-
ZNF1	31780
PRT1	71889
NCP1	31854
NCP2	11927
RPO41	69156
BSP2	-
ETF1	71892
STE11	32039
MYO2	63174
BSP3	12167
IKS1	32011
RPL39	31788
LPD1	39666
CID1	39663
BSP1	63193
RUM1	63213
GEF1	39658
CND05620	63080
CND05650	18302
CND05510	31674
CND05500	69090
CNK02480	31766
CNN02200	44456
CND02430	63071
CND05600	57155
CNE04880	31623
CND05350	63081
CND05490	71854
CND05360	31582
CND05440	63083
CND05430	69095
UM00103	34616
SPO14	34686

continued

LACBIDRAFT_300680	69096
CND05530	69097
CND05520	44460
CND05560	71857
CND05540	71858
CND05480	-
CND05550	74134
CNH00610	71878
CNF01610	74136
UM02469	63150
UM04613	63151
CNG04540	39596
CNB00610	39597
CNB00600	74139
CND01560	69149
CND01570	71888
CNH01100	31883
CNE02190	74147
CNG02430	74148
CND01550	44531
CND01540	23695
CNI00160	74150
CNE02670	69163
CNA03170	63178
CNN00870	44537
CND05390	31826
An11g04720	74152
CND05260	39631
UM02602	63184
CND01530	70130
CND01240	39635
CNA05340	-
CND01400	44548
CND01580	31927
LACBIDRAFT_292741	63190
CND01520	44549
CND01640	63211
CND01630	-
RPL22	34691
SXI1	57675
SXI2	65105
