# Supplementary material for: The Mating Type Locus (MAT) and Sexual Reproduction of Cryptococcus heveanensis: Insights into the Evolution of Sex and Sex-Determining Chromosomal Regions in Fungi
Source: PLoS Genet. 2010 May 20;6(5):e1000961. doi: 10.1371/journal.pgen.1000961 (PMC2873909; doi:10.1371/journal.pgen.1000961)
Supplement: Figure S1 — Eight BCC isolates are confirmed to be C. heveanensis, while the strains CBS 9459 and CBS 6097 are closely related but different species. Eight isolates obtained from BCC have identical D1/D2 sequence as the C. heveanensis type strain CBS 569, while there are two differences between CBS 569 and CBS 9459, and three differences between CBS 569 and CBS 6097. Similarly, six of the eight BCC strains have identical ITS sequence as the type strain CBS 569, and there is only one difference between the type strain and the strains BCC 8396 and BCC 11754. However, there are eleven differences between CBS 569 and CBS 9459, and eight differences between CBS 569 and CBS 6097 in the ITS region. (0.82 MB PDF) [file pgen.1000961.s001.pdf]

Figure S1 (A)

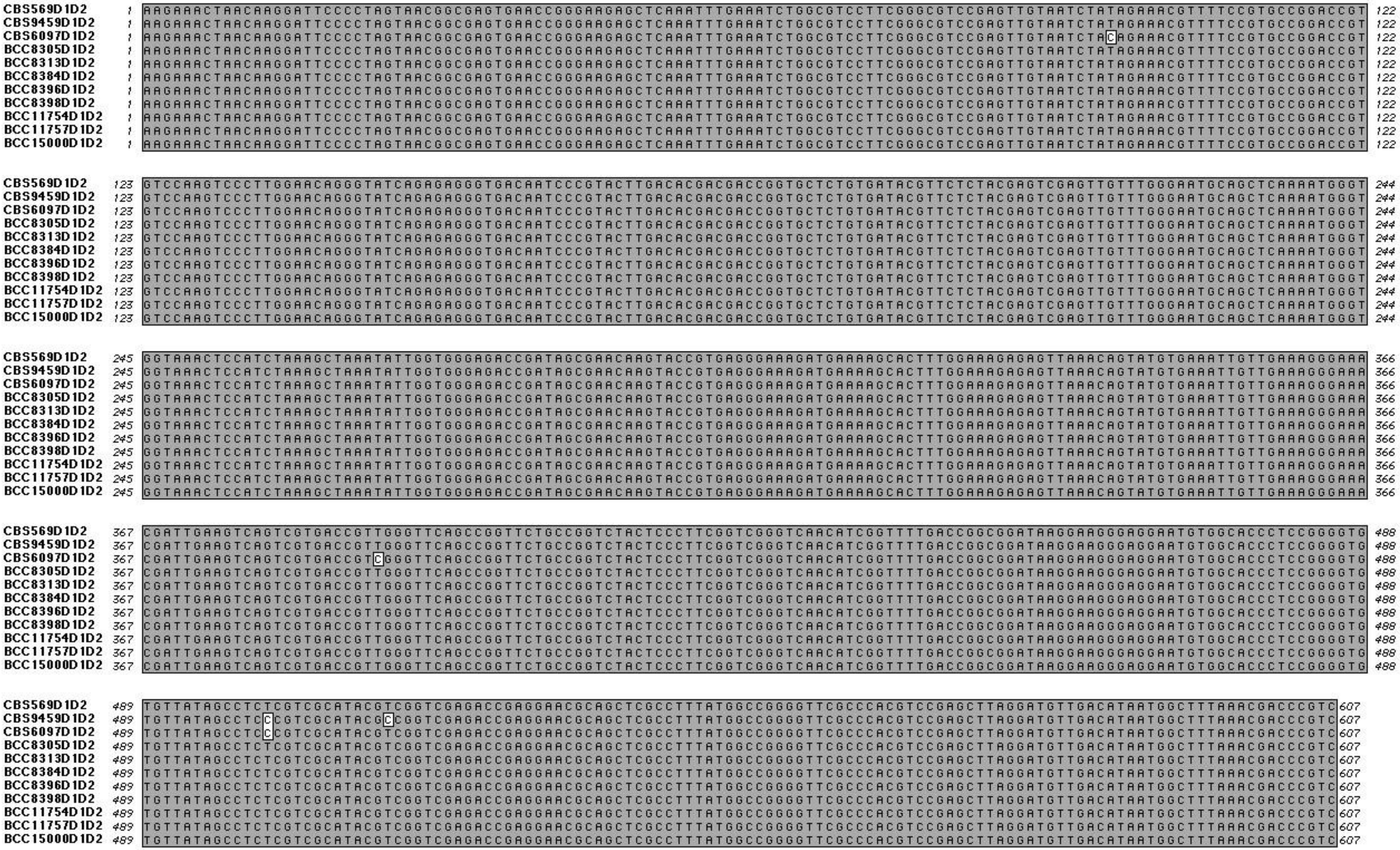

CBS569D1D2
CBS9459D1D2
CBS6097D1D2
BCC8305D1D2
BCC8313D1D2
BCC8384D1D2
BCC8396D1D2
BCC8398D1D2
BCC11754D1D2
BCC11757D1D2
BCC15000D1D2

Figure S1 (B)

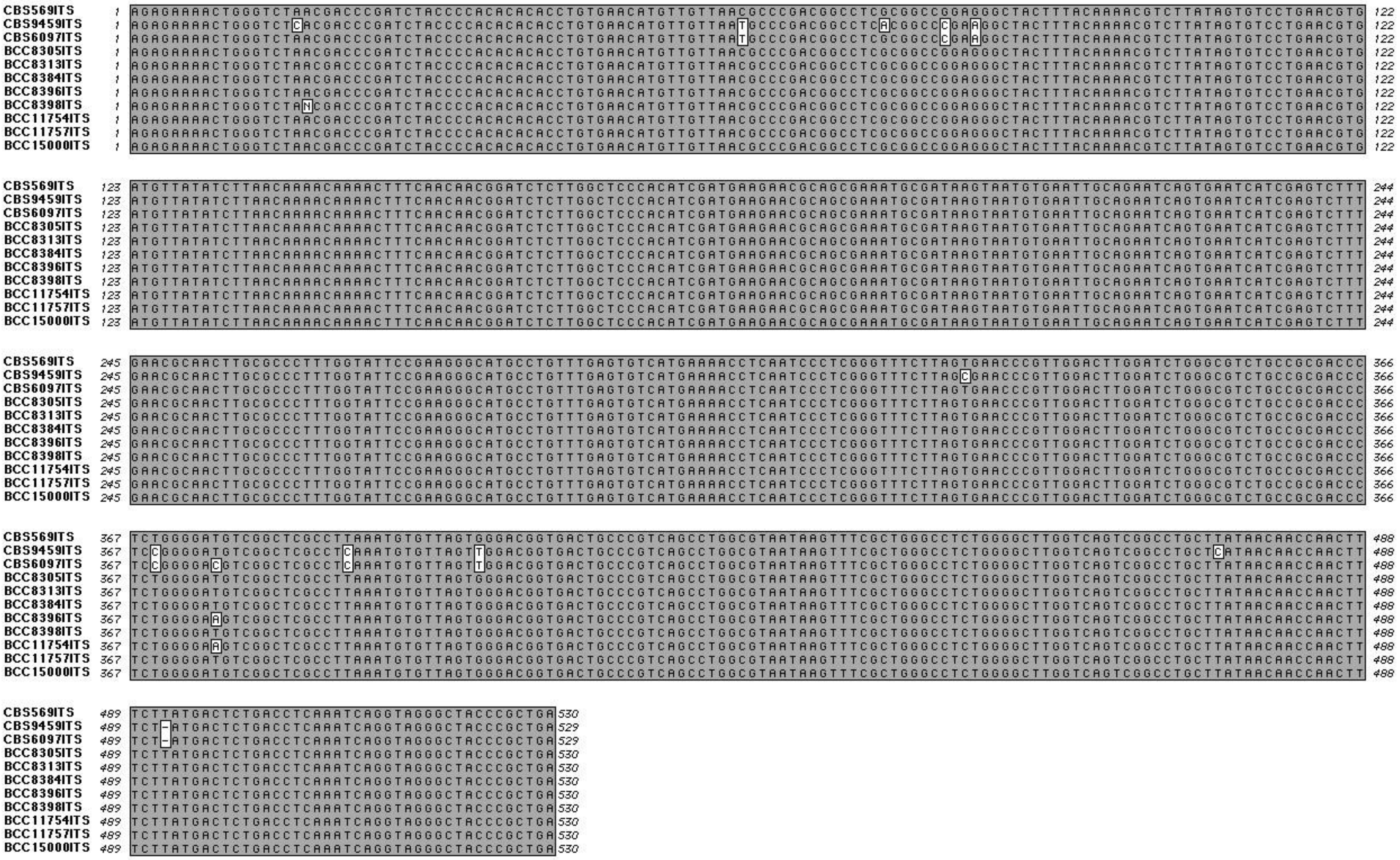

CBS569ITS
CBS9459ITS
CBS6097ITS
BCC8305ITS
BCC8313ITS
BCC8384ITS
BCC8396ITS
BCC8398ITS
BCC11754ITS
BCC11757ITS
BCC15000ITS
